# An Experimental Examination of Demand-Side Preferences for Female and Male National Leaders

**DOI:** 10.3389/fpsyg.2020.576278

**Published:** 2020-09-15

**Authors:** Gregg R. Murray, Bruce A. Carroll

**Affiliations:** ^1^Department of Social Sciences, Augusta University, Augusta, GA, United States; ^2^Georgia Gwinnett College, Lawrenceville, GA, United States

**Keywords:** gender disparity, leadership preferences, experiment, evolution, physical formidability

## Abstract

Females constitute a far smaller proportion of political leaders than their proportion in the general population. Leading demand- and supply side explanations for this phenomenon account for some of the variance but leave a great deal unexplained. In an effort to account for additional variance, this research evaluates the issue informed by the biological theory of evolution by natural selection, a foundational explanation for the diversity and function of living organisms. It experimentally assesses how varying types of inter- and intragroup threat–a recurring ancestral problem–affect demand for female and male national leaders. This work analyzes data collected from individuals (*N* = 826) in the U.S. during the 2012 Cooperative Congressional Election Study. The results suggest the predominant preference for male over female leaders in some contexts may be the non-adaptive and non-functional but lingering outcome of an adaptive preference for physically formidable allies that was shaped by natural selection in ancestral environments.

## Introduction

In the last several decades, women have attained unprecedented success in the electoral arena (Geiger and Kent, 2017). However, the proportion of women attaining political leadership still falls far short of their proportion of the world population. According to a recent review conducted for Pew Research Center (Geiger and Kent, 2017), less than 10 percent of United Nations member states are currently led by a female, and less than 40 percent of the 146 countries examined by the World Economic Forum have had a female head of state or government in the last 50 years. Leading explanations focus on demand-side factors such as role congruity (e.g., Eagly and Karau, 2002) and gender stereotyping (e.g., Huddy and Terkildsen, 1993) as well as supply side factors such as differential political ambition (e.g., Fox and Lawless, 2004). Although these explanations account for some of the imbalance in female-male leadership attainment, they, like most social science models, leave a great deal to explain. This research suggests natural selection, a biologically informed approach widely used outside the social sciences but frequently overlooked within them, may offer additional explanatory leverage on this phenomenon by accounting for differences between females and males in physical formidability.

A cross-disciplinary review of the literature suggests the imbalance in leadership attainment is not surprising. Diverse fields such as non-human animal behavior, anthropology, economics, and psychology document a direct relationship between gender and leadership over millennia and across cultures and species (Murray and Murray, 2011). For instance, with few exceptions (e.g., killer whales, lions, spotted hyenas, bonobos, lemurs, and elephants), male group dominance is nearly universal in primate and mammalian animal groups (Smith et al., 2018; Kappeler et al., 2019). This, of course, includes humans. For instance, Brown (1991, 137) states that “In the public political sphere men form the dominant element among [every people or people in general]” (see also Ludwig, 2002; von Rueden et al., 2018). Archeological and anthropological data suggest that human males have been vastly over-represented in the public sphere dating back thousands of years to Egyptian pharaohs; Chinese, Japanese, and Roman emperors; Catholic Popes; and European monarchs (Murray and Murray, 2011). This includes not just large-scale states or empires but also other forms of society including small-scale, egalitarian, and preindustrial and nonindustrial societies (Whyte, 1978; Collier and Rosaldo, 1981; Low, 1992; von Rueden et al., 2018). Ludwig concludes “this blanket prejudice against female rulers goes back to antiquity” (2002, 29). Moreover, in modern times, the proportion of female political and business executive leaders tops out at about 10 percent (Murray and Murray, 2011; Geiger and Kent, 2017).

Leading explanations for this phenomenon suggest that it results from culturally transmitted, learned stereotypes. Non-human animal, small-scale, egalitarian, nonindustrial, ancient Egyptian, medieval European, and modern corporate cultures are vastly different, though. These cross-temporal, cross-cultural, and, in particular, cross-species results suggest that these unrepresentative leadership outcomes are not due solely to learning stereotypes through cultural transmission. Substantial evidence shows that human behavior is not only affected by environment. It is also affected by biological transmission such as genetic inheritance (Alford et al., 2005; Benjamin et al., 2012; Smith et al., 2012) along with many other biological forces, through time, culture, and species.

The research presented here suggests explanations informed by Darwin’s (1859) evolutionary theory of natural selection may shed additional light on the relationship between gender and leadership preferences. In particular, this argument suggests the imbalance in female-male leadership attainment may be the non-adaptive and non-functional but lingering outcome, or incidental by-product, of an adapted psychological mechanism that favors a preference for physically formidable leaders in certain situations. In short and consistent with an emerging literature on adaptive followership theory (e.g., Little et al., 2007; Van Vugt et al., 2008a; Spisak et al., 2012; Van Vugt and Grabo, 2015; Laustsen and Petersen, 2017), physically formidable leaders may have helped allies acquire and ward off competitors for resources that were vital for survival and reproduction in violent ancestral times (Lukaszewski et al., 2016). Because males have been physically larger than females throughout human history (Geary, 1998) and because humans sometimes rely on “mismatched” cues more suitable for ancestral than modern society (Tooby and Cosmides, 1992; Li et al., 2017), males aspiring to leadership positions in certain situations may have a greater probability of success than females as the result of a by-product of an adaptive preference for physically formidable allies^1^.

To test for effects of evolution-related forces on the demand of followers for female versus male leadership, this article first presents a review of the pertinent literature regarding the role of gender heuristics in leadership preferences and then identifies potential evolutionary factors that may influence leadership preferences within that literature. Next, it presents the results from an experiment (*N* = 826) embedded in the 2012 Cooperative Congressional Election Study (CCES), a nationally representative, population-based online survey that offers the advantage of the external validity of a nationally representative sample and the internal validity of an experimental design. It then presents the results, which suggest that increased intergroup threat increases preferences for physically formidable leaders, which, in turn, increase preferences for male over female leaders. Together, these findings are consistent with the assertion that the preference for male over female leaders may be the incidental by-product of an evolved psychological mechanism. Finally, it ends with a discussion of the implications of the findings, including a possible but controversial policy solution that has been implemented in a number of countries to attempt to overcome gender imbalances in leadership that may be a result of very long-term biological influences.

### Gender, Evolution, and Leadership Preferences

#### Gender-Related Factors

Scholars offer a number of explanations for the imbalance in females and males in political leadership attainment. Broadly speaking, the explanations can be categorized as supply- and demand-side factors (Sweet-Cushman, 2016). Leading supply side factors, which affect the number of female candidates willing to run, include the lack of female role models at elite levels of professional life (e.g., Mansbridge, 1999; Campbell and Wolbrecht, 2006), differential political ambition (e.g., Fox and Lawless, 2004; see also Campbell, 1999), and family role commitments (e.g., Sapiro, 1982; Fulton et al., 2006), which include biological factors related to sexual reproduction (gestation and lactation; Brown, 1991; Low, 1992; Campbell, 1999, 2013; Benenson, 2013; Garfield et al., 2019). Key demand-side factors, which motivate followers’ leadership preferences, include gender stereotyping (e.g., Huddy and Terkildsen, 1993; Kahn, 1996; Sanbonmatsu, 2002; Bauer, 2015), media treatment (e.g., Kahn, 1992, 1996; Devitt, 2002), and political recruitment (e.g., Niven, 2006; Fox and Lawless, 2010; Ashe and Stewart, 2012).

This research is designed to assess followers’ preferences for leaders, so the theoretical argument presented here takes a demand-side approach. Stereotyping, the primary confounding demand-side explanation assessed in this work, suggests that individuals’ evaluations of others are affected by group-related considerations they likely learned to facilitate their understanding and processing of social group-related situations (McGarty et al., 2002). Scholars have argued that gender stereotyping occurs in a variety of situations encompassing such diverse areas as coaching decisions (Kalin and Waldron, 2015), student evaluations of teaching (Centra and Gaubatz, 2000), law firm hiring (Gorman, 2005), career choice evaluation (Correll, 2004), and, most pertinently here, assessments of political leaders (e.g., Sapiro, 1981, 1983; Rosenwasser and Seale, 1988; Alexander and Andersen, 1993; Huddy and Terkildsen, 1993; Matland, 1994; Kahn, 1994; Koch, 2002; Sanbonmatsu, 2002; King and Matland, 2003; Dolan, 2010, 2014; Carey and Lizotte, 2019).

A significant body of literature indicates that voters possess particularized expectations or stereotypes about female and male leaders (e.g., Huddy and Terkildsen, 1993; McDermott, 1998; Eagly and Karau, 2002). Females are generally stereotyped as more communion oriented (Eagly et al., 2019) such as being more compassionate, compromising, emotional, and sensitive. Males are generally stereotyped as more agency oriented (Eagly et al., 2019) such as being more aggressive, assertive, self-confident, and tough (Sapiro, 1981, 1983; Rosenwasser and Seale, 1988; Leeper, 1991; Alexander and Andersen, 1993; Huddy and Terkildsen, 1993; Sanbonmatsu, 2002; Lawless, 2004). In terms of government policy, females are believed to be advantaged on issues such as education, health, and care for children and the elderly, while males are thought to hold an advantage on issues such as crime, the military, and economics (Dolan, 2014).

It is important to note, though, that the effects of gender can be complicated. Scholars have found that gender stereotypes can transcend political party (Plutzer and Zipp, 1996), but in several instances the effect of gender is rendered insignificant when controlling for partisanship, incumbency, or both (Dolan, 2014; Stadelmann et al., 2014). In other situations, gender heuristics have been found to interact with female candidates’ ideological orientation, which, for instance, can hinder conservative female candidates (Sanbonmatsu and Dolan, 2009) where stereotypes regarding women, such as positions on abortion, may handicap them compared to their liberal counterparts.

#### Evolutionarily Informed Demand Factors

The literature provides substantial evidence in support of gender effects associated with stereotyping and other demand-related factors, but there is a great deal of variance left to be explained regarding how gender-based considerations affect leadership preferences. Research shows that biological factors also contribute to human behavior (e.g., Chagnon and Irons, 1979; Scarr and McCartney, 1983; Bouchard, 2004; Faulkner et al., 2004; Iofrida et al., 2014; Manuck and McCaffery, 2014), including political behavior (e.g., Alford and Hibbing, 2004; Alford et al., 2005; Kanai et al., 2011; Arceneaux et al., 2012; Hatemi and McDermott, 2012; Aarøe and Petersen, 2013; Merolla et al., 2013; Adams et al., 2014; Dawes et al., 2014, 2015; French et al., 2014; Shah et al., 2015; Stewart et al., 2015; Klofstad, 2016; Murray, 2017; Weinschenk and Dawes, 2017). The research presented here suggests that another key effect, evolution by natural selection, a foundational explanation for the diversity and function of living organisms, may explain additional variance. The application of its general principles may account for how leadership preferences vary by leadership situations (e.g., Van Vugt and Spisak, 2008; Post, 2015) that are evolutionarily relevant. It is appropriate to note, as stated by a reviewer of this article, “the theory of evolution by natural selection makes no predictions whatsoever about leadership preferences…[it] only provides some very general principles that can be applied in many specific ways to understand the evolution of particular traits.” Put otherwise, while the theory of evolution holds substantial explanatory powers for matters involving biological systems, it is important to be clear that the following leadership-specific hypotheses are informed by but not derived directly from evolutionary theory.

Evolution by natural selection suggests that physical, cognitive, emotional, and motivational mechanisms emerged because they resulted in a greater likelihood of an individual’s survival and ability to reproduce (e.g., Mayr, 2001; Crawford, 2008). Given the typically slow speed of evolution, the human brain, like other parts of the human body, still reflects the hominids living in the environment of evolutionary adaptedness (Tooby and Cosmides, 1992; Foley, 1995). Instincts acquired through natural selection in human ancestral times manifest themselves in modern life, even when seemingly irrational in and mismatched to the context of modern living (e.g., Li et al., 2017; Giphart and van Vugt, 2018). For instance, the widespread fear of snakes (LoBue and DeLoache, 2008; Hoehl et al., 2017), which are rarely encountered in contemporary society, and the overconsumption of fatty and sweet foods (Nesse and Williams, 1994), which promoted survival in times when adequate nutritional intake was uncertain, continue today despite their mismatches to modern society.

Similarly, the probability that a modern national leader will physically lead troops into battle is extremely small. But prior research suggests that individuals prefer physically formidable political leaders, a preference some scholars suggest is due to evolutionarily shaped preferences regarding physically formidable allies helping others acquire and protect vital resources in evolutionary environments (Murray, 2014; von Rueden et al., 2014; Lukaszewski et al., 2016). This suggests that individuals discount the aspects of modern society that render characteristics like size irrelevant and can make leadership decisions using cues that were suitable to older, small-scale societies (Little et al., 2007).

The human species has lived roughly 99 percent of its existence in small hunter-gatherer communities of roughly five to 150 people (Diamond, 1999; Foley, 1995; Van Vugt et al., 2008b). Intra- and intergroup conflict were common (e.g., Chagnon, 1997; Keeley, 1996; Van Vugt et al., 2008b) as individuals and groups competed over resources and status related to survival and reproduction. In terms of applying an evolutionary analytical framework (Lewis et al., 2017), this competition created a frequent and impactful adaptive problem regarding threats to individual survival and growth in the form of aggression and conflict initiated by competitive and/or dangerous conspecifics over vital resources such as food, shelter, and social status (Petersen et al., 2008).

One potential solution to such dangerous ancestral environments was physically formidable leaders who, in the pursuit of prestige and the related benefits (e.g., Henrich and Gil-White, 2001), helped allies offensively acquire and defensively protect vital resources due to their significant resource holding potential [regarding offensive versus defensive leadership see Laustsen and Petersen (2017) and Lukaszewski et al. (2016)]. Some scholars note that leadership in ancestral times was gained through capabilities that included fighting skills and strength (Diamond, 1999; Van Vugt et al., 2008a). For instance, leaders were called on by followers to quell intragroup fights and to lead raids against adversary groups (Van Vugt et al., 2008a). As succinctly summarized by Lukaszewski et al. (2016, 385): “Ancestrally, physically formidable males would have been differentially equipped to generate benefits for groups by providing leadership services of within-group enforcement… and between-group representation…” Intra-group enforcement might include punishing free riders and rule breakers, intervening in fights and other conflict between group members to reduce group-threating disputes, and enforcing group coordination to keep members on task. On the other hand, between-group coordination might include engaging in face-to-face negotiations with other groups and serving in war in times of extreme conflict (Lukaszewski et al., 2016).

Modern leadership preferences reflect these ancestral forces through a number of characteristics. First, there is substantial evidence of a biological component, particularly genetic inheritance, to political behavior (e.g., Alford et al., 2005) and leadership attainment (e.g., De Neve et al., 2013; Oskarsson et al., 2017). This evidence lays a solid foundation for related biological factors like evolutionary forces to play a role in leadership preferences. Second, in social interactions, individuals establish hierarchies quickly based on perceived authority, even using first impressions that can occur before any verbal interaction (Kalma, 1991). Importantly, humans have the ability to evaluate visually a person’s physical formidability (Sell et al., 2008). Third, in leadership preferences, the context matters as different leadership situations require different leader responses (McCleskey, 2014). For example, research suggests female leaders more effectively coordinate large teams and cultivate team cohesion and communication (Post, 2015). This is consistent with findings that female leaders are strongly preferred and more successfully raise group investment than male leaders during intragroup competition (Van Vugt and Spisak, 2008). On the other hand, male leaders more successfully raise group investment during intergroup competition and, more broadly, people tend to prefer more dominant leaders when the chance of danger increases (McCann, 2001; Little et al., 2007; Merolla et al., 2007; Petersen and Laustsen, 2020).

Fourth, this is consistent with prior findings that individuals with greater physical stature, as indicated by relative height, are more likely to be perceived as capable and competent (Hensley, 1993) and to be respected and feared by potential opponents (Gregor, 1979). To extend this, research also suggests people are less likely to aggress against opponents who are physically formidable (e.g., Fessler et al., 2014). Broadly speaking, formidability is defined as the ability to hold resources by imposing costs on challengers (Sell et al., 2008). Physical size is an effective indicator of formidability related to fighting ability. Larger animals, both human and non-human, are more likely to prevail in physical contests (e.g., Huntingford and Turner, 1987; Sell et al., 2012; Szamado, 2008), and, therefore, individuals frequently use physical size as an indicator of resource holding potential (Huntingford and Turner, 1987).

It is theoretically important not to conflate dominance with physical formidability. They are different concepts, and this research specifically addresses physical formidability. Dominance has been defined as, for instance, “the induction of fear, through intimidation and coercion” (Petersen and Laustsen, 2020, 136). Physical formidability as used here is indicated by physical characteristics (e.g., height, weight, body mass index) that can be used “to hold resources by imposing costs on challengers.” A physically formidable person may or may not induce fear (i.e., be dominant); the person may merely cue that in the case of a physical altercation he or she will have an advantage over an opponent. But when a physically formidable individual does induce fear, it is because the opponent believes it is likely he or she will be physically harmed or “beat up.” On the other hand, a dominant person is by definition inducing fear. Importantly, though, that person may induce fear because of physical formidability or myriad other reasons. The person may be brandishing a lethal weapon or holding a position of social advantage such as a powerful role in an organization (e.g., a supervisor of other people) or possessing resources that could damage personal, social, or professional reputations (e.g., social or news media). Put otherwise, although the two sometimes go together, one can be dominant without being physically formidable, and one can be physically formidable without being dominant.

This argument is in line with evidence that war stimulates a preference for leaders with greater weight and body mass (Murray, 2014). This is also consistent with emerging research on adaptive followership theory, which suggests that modern followership preferences are influenced via factors related to natural selection by the outcomes of leadership in ancestral situations of social conflict (Little et al., 2007; Van Vugt et al., 2008b; Spisak et al., 2012; Van Vugt and Grabo, 2015; Laustsen and Petersen, 2017). This review suggests:

Hypothesis 1: Situations of increased intergroup threat will lead to an increased preference for a physically formidable leader.

The above argument and supporting evidence suggest there are adaptive psychological tendencies unrelated to modern gender stereotypes that affect individual preferences in terms of both intra- and intergroup competition and physically formidable leaders. But the connection to preferences regarding gendered leadership requires further evidence and the role of evolution by natural selection requires further specification. Evolution produces three outcomes: adaptations, incidental by-products, and random effects (e.g., Buss et al., 1998; Lewis et al., 2017). Adaptations emerged because they helped solve a recurring problem related to survival and reproduction in ancestral environments. For example, umbilical cords carry nutrition from mothers to their developing fetuses. Based on the argument presented above, this research asserts that the preference for physically formidable allies and leaders is, like umbilical cords, an adaption. Such a preference promoted survival and reproduction as formidable allies helped individuals acquire and protect vital resources. On the other hand, by-products emerged as an outcome of an adaptation. They promote neither survival nor reproduction but accompany adaptations, which do. That is, they are non-adaptive and non-functional such as navels being the results of umbilical cords. This research asserts that the preference for male over female leaders in threatening situations is, like navels, a by-product of evolution. This phenomenon accompanies the preference for physically formidable leaders and does not promote survival or reproduction. Finally, evolution can also produce random effects, which emerged as the result of random or sudden changes in the environment and which are not linked to features of an adaptation. For example, the shape of an individual’s navel is a random effect of evolution that neither helps nor hinders the adaptive function of umbilical cords. This research asserts the psychological mechanism presented here is a by-product of evolution and not a random effect of evolution.

With the mechanism for the evolutionary link specified, the link to gendered leadership preferences can also be specified. Continuing the enumeration from above, fifth, archeological evidence suggests that males have been physically larger than females in all human hominid groups dating back three to four million years (Geary, 1998). This translates in current times to men having on average 61 percent more muscle mass (Lassek and Gaulin, 2009) and roughly 50 to 100 percent more upper-body strength than women (Pheasant, 1983), with female and male distributions in upper-body strength and muscle mass overlapping by less than 10 percent (Lassek and Gaulin, 2009). This sexual dimorphism suggests that when physical formidability is a desirable trait, males are greatly advantaged over females. Sixth, evidence suggests that throughout history males have been more likely to serve as combatants in wars and other intergroup conflict than females (Keegan, 1993; Goldstein, 2003; Glowacki et al., 2017). This is consistent with research that indicates male leaders are strongly preferred over female leaders and more successfully raise group investment than female leaders during intergroup competition (Van Vugt and Spisak, 2008). It is also consistent with research that shows groups with greater numbers of males are more likely to win intergroup contests (Glowacki et al., 2017). This review suggests:

Hypothesis 2: A preference for a physically formidable leader will lead to an increased preference for a male leader compared to a female leader.

If the results support Hypotheses 1 and 2, the next step is to provide evidence that demonstrates the potential role of evolutionary forces in gendered leadership preferences by establishing a link from intergroup threat through preferences for leader physical formidability to preferences regarding the biological sex of a preferred leader. Evidence suggests that part of the male advantage in leadership attainment is related to males’ greater body size and physical strength (e.g., Handwerker and Crosbie, 1982; Glowacki and von Rueden, 2015; von Rueden et al., 2018; but see Low, 1992). Overall, the analyses presented here suggest:

Hypothesis 3: The preference for a male versus female leader will be at least partially attributable to a sense of external threat that is conveyed through a preference for a physically formidable leader.

### Plan of Analysis

**FIGURE 1:**
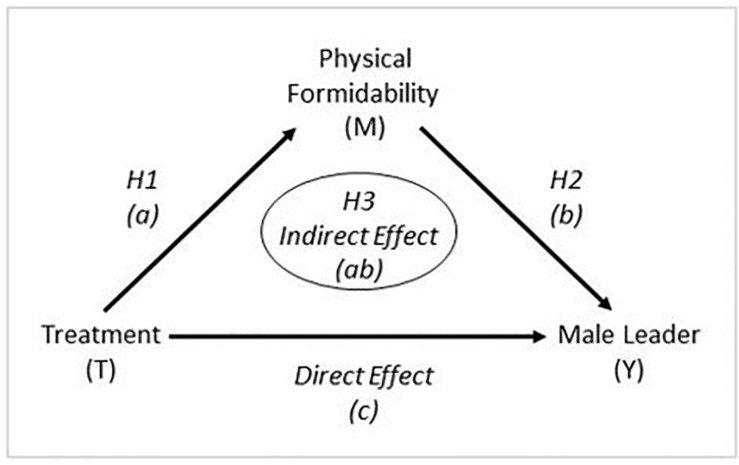
Visual Depiction of the Analysis.

We assert that differential preferences for female versus male leaders are motivated at least partially by situational threat that may be related to evolutionary forces. Increased intergroup threat, an evolutionarily salient situation, increases preferences for physically formidable leaders, and, in turn, a preference for physically formidable leaders increases preferences for male compared to female leaders. To assess this argument and process, we use simple mediation analysis (e.g., Baron and Kenny, 1986; Preacher and Hayes, 2004) to see if a preference for a physically formidable leader contributes to (i.e., mediates) the relationship between the experimental treatments and the preference for a female versus male leader. We first present results of the underlying experiment; that is, did the experimental conditions affect the outcome variable, the preference for a female versus male leader? In terms of mediation analysis this represents the total effect of the relationship between the treatments and leadership preferences; that is, the relationship between the treatments and leadership preferences without controlling for the effect of the mediating variable. Then we turn to the main argument and evaluate evidence regarding intergroup threat and whether it increases preferences for physically formidable leaders (the mediator), testing Hypothesis 1 (H_1_). Next, we assess evidence regarding preferences for physically formidable leaders and whether they increase preferences for male relative to female leaders, testing Hypothesis 2 (H_2_). Finally, we assess evidence regarding whether a differential preference for a female versus male leader is linked to perceived intergroup threat through the preference for a physically formidable leader, the mediator representing the indirect effect (Preacher and Hayes, 2004), testing Hypothesis 3 (H_3_). We assert that supporting evidence for the three hypotheses would provide nontrivial evidence that differential preferences for male versus female leaders are motivated at least in part by situational threat related to evolutionary forces. As depicted in Figure 1, specifically we are testing for the presence of an indirect effect *(ab)* from the experimental treatments (T) through the mediating variable, preference for physical formidability (M), to the outcome variable, leader preference (Y).

## Data and Methods

The data were collected before the 2012 presidential election as part of that year’s Cooperative Congressional Election Study (CCES), an ongoing series of nationally representative, population-based online surveys administered by YouGov/Polimetrix. One thousand subjects participated in the survey experiment with completed responses obtained from *N* = 826 subjects. Compared to population means reported by the United States Census Bureau, the experimental subject pool is slightly more female, racially diverse, and educated; slightly less Hispanic; and similar in terms of wealth. Overall, this research takes advantage of the internal validity offered by an experimental design and the external validity offered by a nationally representative sample. All *p*-values are based on two-tailed tests.

Regarding the experiment, the CCES survey used simple random assignment to assign subjects to one of four treatment groups. The treatments were vignettes directing subjects to “[c]reate in your mind the national leader of your country, such as a president or prime minister, whom you would want to lead the country” during times of varying threat conditions: war, peace, natural disaster requiring cooperation, and a non-specific control condition. This vignette approach (Schoenberg and Ravda, 2000) was used to lead subjects to fix their leader’s characteristics in their minds before answering the follow-up questions regarding specific characteristics that may have led them to change their answers (e.g., some subjects may not have imagined a female leader until a question led them to do so). The war vignette served as the threat condition, while the peace, cooperation, and control vignettes served as reduced-threat conditions. See Supplementary Appendix A for the treatment vignettes. After treatment, the instrument directed subjects to describe in their own words the leader they imagined and then to answer a series of open- and closed-ended questions related to leader preferences stemming from the treatments followed by a series of political and demographic questions.

A multinomial probit test of random assignment to the experimental groups indicates the randomization process generated statistically equivalent experimental groups (*X*^2^[69] = 47.38, *p* = 0.98). In this test, group assignment was regressed on subject gender stereotyping (discussed below), political ideology, income, education, race, gender, age, religiosity, and political interest. See Supplementary Appendix B for details. Manipulation checks indicate the treatments successfully influenced subjects’ assessments of the differences in threat presented by the treatments.

## Results

### The Underlying Experiment: The Total Effect

**FIGURE 2:**
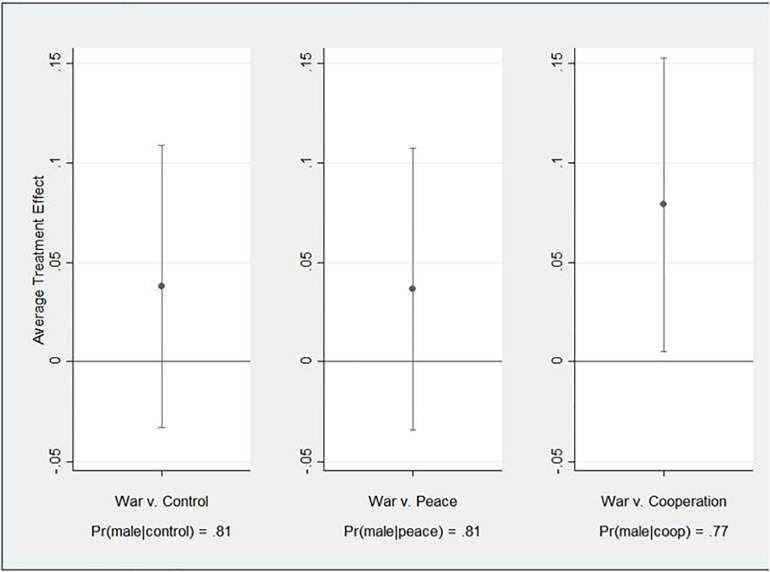
Average Treatment Effects (Total Effects).

This preliminary analysis tests for a relationship between each treatment and the preference for a female versus male leader without controlling for the proposed mediating effect. More formally, this is the total effect *(c)* (Baron and Kenny, 1986; Preacher and Hayes, 2004). As such, subjects responded to a closed-ended question about the gender of their imagined leader, with “male” responses coded 1 and “female” coded 0. This dichotomous measure served as the dependent variable for this and later analyses related to H2 and H3. For these analyses, leader gender was separately regressed on three different independent variables representing the treatment conditions such that subjects in the “war” group were coded 1 and each of the others coded 0. Due to the dichotomous nature of the dependent variable, the effects were estimated using probit regression. Further, they were also estimated using robust standard errors due to evidence of heteroscedasticity in some of the models. Because random assignment was successful, the model does not specify covariates. The probit estimates and statistics for the three models appear in Supplementary Appendix C. Overall, the probability of a preference for a male leader ranges from 0.77 in the cooperation condition (Pr(male| coop)) to 0.81 in the control (Pr(male| control)) and peace (Pr (male| peace)) conditions. For ease of interpretation, Figure 2 presents the average treatment effects in the form of average marginal effects, which are derived from the probit estimates. The figure suggests that moving from the control and peace treatments to the war treatment increases the preference for a male leader but not in statistically discernible ways. On the other hand, the war treatment relative to the cooperation treatment statistically significantly increases the preference for a male leader by 7.9 percentage points or about 10 percent.

Because a total effect is not essential for finding indirect effects in mediation analysis (Preacher and Hayes, 2004; Hayes, 2017), the next steps are to continue working through the mediation analysis process to test the hypothesized indirect effects.

### Testing H_1_: Intergroup Threat and Preferences for Physically Formidable Leaders

The argument presented here indicates intergroup threat leads to a mismatched preference for a physically formidable leader as a result of lingering evolutionary effects on people’s behavior. Specifically, Hypothesis 1 states that increased intergroup threat will lead to an increased preference for a physically formidable leader. If the evolutionarily argument is correct, experimentally stimulated intergroup threat should increase subjects’ preference for a physically formidable leader despite the fact that modern national leaders are extremely unlikely to lead troops into battle.

For the analyses, the subjects assessed the physical formidability of their imagined leaders using a 1–7 scale indicating how well the 10 words or phrases presented in Table 1 described their leader. For all the measures, 1 indicated the word or phrase described their leader “not well at all” and 7 indicated “extremely well.” For these analyses, the leader descriptions are regressed on the treatments, where 1 indicates the war condition and 0 indicates either the control, peace, or cooperation conditions, using OLS estimation. The estimates are based on robust standard errors due to evidence of heteroscedasticity in some of the models. Because random assignment was successful, the models do not specify covariates. Supplementary Appendix D reports the 30 models.

**TABLE 1:** Treatment Effects: Treatments Stimulate Preferences for Leader Characteristics (H1).

	War v.			
	
DV	Cntl	Peace	Coop	DV Mean (SD)
Athletic	0.015	0.016	0.161	4.8 (1.5)
Attractive	−0.012	−0.077	0.164	4.6 (1.5)
Competent	0.092	0.032	0.121	6.4 (1.1)
Dependable	0.085	0.052	0.085	6.5 (1.0)
***Dominant***	*0.244+*	0.400**	0.424**	4.9 (1.5)
Friendly	−0.074	−0.168	−0.063	6.0 (1.2)
Intelligent	0.158	0.039	0.140	6.5 (1.0)
Physically Fit	0.162	−0.073	0.141	5.5 (1.4)
***Physically Impos/Intim***	0.436**	0.362*	0.469**	3.8 (1.8)
*Physically Strong*	0.198	0.031	*0.255+*	5.0 (1.5)
Scales				
***Physical Formidability***	0.328*	0.215	0.367**	4.4 (1.4)
Classic Ldshp	0.117	0.055	0.116	6.5 (1.0)

Dependent Variables (DV) coded 1 = “not well at all” to 7 = “extremely well”; +*p* < 0.10 *p < 0.05 **p < 0.01 ***p < 0.001 (two tailed). Bold indicates measures with multiple statistically significant treatment effects.

Table 1 presents the OLS coefficients or average treatment effects estimated by the models (Figure 1, *(a)*). Of the 10 characteristics, only three generated statistically meaningful effects: physically imposing/intimidating, dominant, and physically strong. The positive and statistically significant effects on the physically imposing/intimidating dependent variable indicate that the war treatment stimulated a meaningfully greater preference for a physically imposing/intimidating leader compared to each of the other treatments. The magnitude of these effects is not trivial. The threatening war treatment increased the preference for a physically imposing leader relative to the control and peace treatments by about 0.4 point or 10 percent and the cooperation treatment by about 0.5 point or 13 percent. The war treatment also increased the preference for a dominant leader relative to the peace and cooperation treatments by about 0.4 point or 8.5 percent. Further, it had a positive effect on preference for a dominant leader relative to the control treatment but only reached a marginal level of statistical significance. Finally, the war treatment also had a positive effect relative to the cooperation treatment on the preference for a physically strong leader but only reached a marginal level of statistical significance. The measures “physically imposing or intimidating” and “physically strong” were intended to represent physical formidability. The preference for a physically strong leader is not as clear as it is for a physically imposing/intimidating leader, but the results are mostly consistent with arguments that increased intergroup threat stimulates a preference for physically formidable leaders. Overall, these results provide reasonable evidence in support of H_1_.

Interestingly, the war treatment did not stimulate a discernible effect on any of the classically preferred leadership characteristics (i.e., competent, dependable, and intelligent; e.g., Miller et al., 1986; Zaccaro et al., 2004). This is likely a reassuring result given that it indicates individuals value these traits regardless of the context, which is also indicated by their high mean scores (minimum of 6.4 out of maximum possible of 7) reported in Table 1 for these characteristics. Together these three measures of classic characteristics create an internally consistent scale in these data with Cronbach’s alpha = 0.91. Table 1 indicates the war treatment also had no statistically discernible effect on this scale of classic leadership traits compared to the non-threat treatments.

To specifically assess physical formidability, the two measures physically imposing/intimidating and physically strong are used to create a measure of physical formidability that constitutes a reasonable scale, particularly for only two items, with Cronbach’s alpha = 0.59 and a moderate bivariate correlation *r* = 0.43 (*p* < 0.001). In the case of this scale, Table 1 indicates the war treatment meaningfully increased relative to the control and cooperation treatments the preference for a physically formidable leader. It increased the preference for a physically formidable leader by 0.3 point or 7 percent relative to the control treatment and 0.4 point or 9 percent relative to the cooperation treatment. Overall and again, these results mostly support H_1_. They are consistent with arguments that increased intergroup threat triggers a preference for physically formidable leaders.

### Testing H2: Preferences for Physically Formidable Leaders and Leader Sex

Having provided evidence in support of H_1_ that intergroup threat stimulates a preference for physically formidable leaders, the second set of analyses is designed to establish a relationship between a preference for a physically formidable leader and the sex of the leader. In particular, H_2_ states that a greater preference for a physically formidable leader will lead to an increased preference for a male leader compared to a female leader. If this argument is correct, then subjects’ leader preferences should account for the biological condition and everyday experience of sexual dimorphism in which human males tend to be larger and more physically formidable than human females.

To test this hypothesis, leader gender was regressed on the physical formidability scale created for H_1_ as well as a measure of gender stereotyping and a number of covariates found in previous research to affect attitudes toward differential preferences for female versus male leaders: subject’s sex, age, education, religiosity, and political ideology. Education, religiosity, and ideology were specified as series of indicator variables as noted in Table 2. Physical formidability was recoded to a 0–1 scale to facilitate comparison with other measures. The measure of gender stereotyping is a “multidimensional aversion to women who work scale,” which estimates skepticism of female employment and traditional role preferences (Valentine, 2001). The analysis includes this measure to control for leadership preferences motivated by attitudes toward gender equality at work. Stereotyping is the primary alternative explanation to the evolutionary argument presented here. This 10-item scale (Cronbach’s alpha = 0.91) represents learned or environmental effects on this leadership preference, and the expectation is that it will exert an independent effect on the gender of the imagined leader such that individuals with a greater “aversion” to women at work will be more likely to prefer a male leader. This scale was recoded to range between 0 and 1 to facilitate comparisons with the measure of preferences for physical formidability. In particular, if effects of the physical formidability measure on the sex of respondents’ preferred leader disappear when this measure is included in the model, then we can conclude that it is an effect of stereotyping and that evolution-related forces, at least as construed here, do not affect this leadership preference. See Supplementary Appendix E for details on pertinent variables.

**TABLE 2:** Average Marginal Effects: Preference for Male (v. Female) Leader.

	AME (1)	AME (2)	AME (3)	AME (4)
Physical Formidability	0.325***	0.320***	0.307***	0.300***
Stereotyping		0.310***	0.217**	0.121
Female			−0.090**	−0.084**
Age			0.002*	0.001
Education				
- high school or less			–	–
- some college			−0.046	−0.044
- college degree			−0.000	0.005
Religion Important				
- great deal			–	–
- somewhat			0.046	0.066+
- not much			−0.028	0.002
- not at all			−0.096*	−0.048
Ideology				
- liberal				–
- moderate				0.054
- conservative				0.154***
- not sure				−0.045
Probit Model				
N	826	826	826	826
*X*^2^	26.87***	35.20***	74.73***	97.96***
Pseudo *R*^2^	0.04	0.07	0.11	0.14

Dependent variable: coded 1 = male and 0 = female. – indicates omitted category. +*p* < 0.10 *p < 0.05 **p < 0.01 ***p < 0.001 (two tailed).

Supplementary Appendix F presents the full regression models, which use probit estimation due to the dichotomous nature of the dependent variable and robust standard errors due to evidence of heteroscedasticity. For ease of interpretation, Table 2 presents the average marginal effects for the four probit models in order to demonstrate the effect of subjects’ preferences for leader physical formidability on their preferences for a female versus male leader (Figure 1, *(b)*). The first column of results indicates that moving from the minimum to maximum value of leader physical formidability increases the probability of subjects preferring a male leader by 32.5 percentage points (*p* < 0.001, 95% CI [20.7, 44.3]). The second column shows that including the control for gender stereotyping in the model only trivially reduces the marginal effect of physical formidability from 32.5 percentage points to 32.0 percentage points (*p* < 0.001, 95% CI [20.1, 43.8]). It is also worth noting that moving from a subject who stereotypes the least to one who stereotypes the most increases the probability of preferring a male leader by 31.0 percentage points (*p* < 0.001, 95% CI [15.3, 46.6]).

Columns 3 and 4 show that including pertinent socio-demographic and political covariates only trivially attenuates the effect of leader physical formidability on preferences for a male leader. The socio-demographics decrease the effect by slightly more than one percentage point to 30.7 percentage points (*p* < 0.001, 95% CI [19.3, 42.0]), while decreasing it by less than one percentage point to 30.0 when political ideology is also included (*p* < 0.001, 95% CI [19.0, 41.1]). It is worth noting that the effect of gender stereotyping decreases substantially across the range of models declining to 21.7 percentage points (*p* = 0.01, 95% CI [6.6, 36.9]) when the socio-demographics are also included and to a statistically insignificant effect (12.1, *p* = 0.12, 95% CI [−2.9, 27.1]) when political ideology is included as well.

The effect of preferences for leader physical formidability on preferences for female versus male leaders persists across a number of models that include pertinent controls including gender stereotyping, the primary alternative explanation. Further, the effect is only trivially attenuated as the controls are added, dropping from a 32.5 percentage-point effect in the bivariate model to a 30.0 percentage-point effect in the fully specified model. These results support H_2_. They are consistent with arguments that the preference for a physically formidable leader is associated with a decreased preference for a female leader.

### Testing H3: Threat Affects Leader Preferences Through Physical Formidability

Having provided evidence that intergroup threat stimulates a greater preference for physically formidable leaders (H_1_) and demonstrated that a greater preference for a physically formidable leader stimulates a greater preference for a male leader (H_2_), the third and final set of analyses is designed to establish a link from intergroup threat through preferences for leader physical formidability to preferences regarding the biological sex of a preferred leader (Figure 1, *(ab)*). Specifically, H_3_ states that the preference for a male versus female leader will be at least partially attributable to a sense of external threat that is conveyed through a preference for a physically formidable leader. This analysis is intended to test the key link between the evolutionarily salient treatments and preferences for male versus female leaders.

This study uses causal mediation analysis to test this hypothesis. Causal mediation analysis is designed to “quantify the effect of a treatment that operates through a particular mechanism…the key quantity of interest is the calculation of how much of the treatment variable is transmitted by the mediating variable” (Hicks and Tingley, 2011, 606). In this study, causal mediation models link each threat stimulus with preferences regarding sex of the preferred leader through preferences for a physically formidable leader.

**FIGURE 3:**
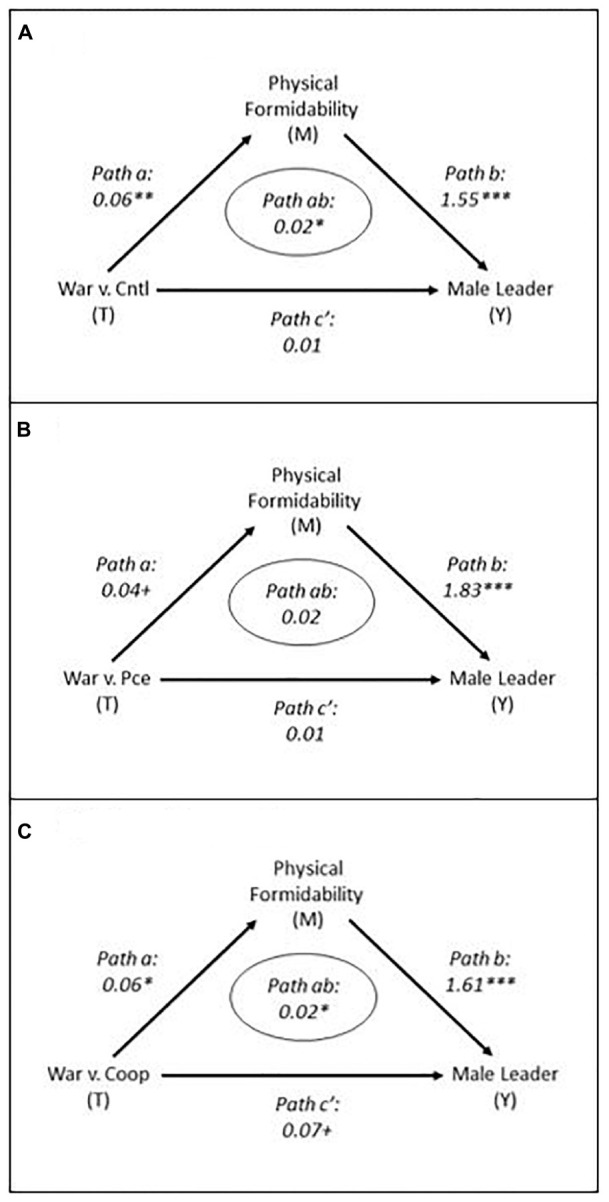
Mediation Models Indicating Indirect Effects.

Figure 3 presents the formal causal mediation models. Supplementary Appendix G presents the full models. The effect of the threat stimulus on the preference for a physically formidable leader (path *a*, the quantitative estimate of Figure 1, *(a)*) and the effect of the preference for a physically formidable leader on the preference for a male leader (path *b*, the quantitative estimate of Figure 1, *(b)*) constitute the indirect effect from the threat stimulus to leadership preference (path *ab*, the quantitative estimate of Figure 1, *(ab)*). Path *ab*, the indirect effect, is the effect of primary interest. Path *a* was estimated with OLS linear regression specifying a bivariate model regressing the preference for a physically formidable leader on the specified treatment. This is the relationship established in tests of H_1_. Path *b* was estimated with probit regression specifying a multivariate model regressing leader sex preference on leader physical formidability preference, the specified treatment, gender stereotyping, and several socio-demographic and political covariates included in the test of H_2_ (i.e., respondent biological sex, age, education, religiosity, and political ideology). This is the relationship established in tests of H_2_. For completeness, the models report the direct effect (path *c’*) of the treatments on the preference for a male leader after controlling for the indirect effect (path *ab*). As a reminder, this is not the same as the total effect, which does not control for the mediated effect, discussed above. Path *c’* was estimated in the same model as path *b*. All paths were estimated using the Mediation package in Stata (Hicks and Tingley, 2011) and robust standard errors.

The figure presents the primary effects of interest, threat on preferences for a male versus female leader via preferences for a physically formidable leader (i.e., path *ab* or the indirect effects). These results demonstrate the expected effects in two of the three cases. They indicate that compared to respondents receiving the non-threat treatments, those who received the threat treatment of war compared to the control (Figure 3 panel A) and cooperation (Figure 3 panel C) treatments were statistically more likely to prefer a male leader, and this preference was partially attributable to a greater preference for a physically formidable leader. The results also hint at an indirect effect of war compared to peace (*p* = 0.104 two tailed).

These results mostly support H_3_. They indicate that intergroup threat tends to stimulate a greater preference for a male versus female leader, and that greater preference is partially transmitted through the preference for a physically formidable leader.

## Discussion

Research in a variety of contexts finds that individuals often use gender-based heuristics to evaluate females and males. Learning or environment-related explanations such as gender stereotyping (e.g., Huddy and Terkildsen, 1993; Kahn, 1996; Sanbonmatsu, 2002; Bauer, 2015) successfully account for some of the variance in this behavior, but, like most social science models, they also leave a substantial amount of variance to explain. Because human behavior is the result of both environment and biology and the interaction between the two, this research attempts to account for additional variance by broadly looking at the issue employing the biological theory of evolution by natural selection, a foundational explanation for the diversity and function of living organisms, and by specifically framing leadership preferences in terms of an evolutionary consideration, varying levels of group threat. The results for tests of H_1_ generally suggest that increased group threat increases preferences for physically formidable leaders. In particular, increased threat increases the preference for a physically imposing/intimidating leader and to a lesser degree a physically strong leader. The results clearly support H_2_ and indicate that the preference for a physically formidable leader is associated with an increased preference for a male leader. Finally, two of three tests of H_3_ indicate there is a causal link between increased intergroup threat and the preference for a male over female leader that is at least partially attributable to subjects’ preference for a physically formidable leader.

Overall, the results support the argument that the advantage males have over females in regard to national executive leadership may be the result of long-term evolutionary forces. In terms of applying an evolutionary analytical framework (Lewis et al., 2017), the ancestral environment posed a frequent and impactful adaptive problem of threats to individual survival and growth in the form of aggression and conflict over vital resources such as food, shelter, and social status (Petersen et al., 2008). One potential solution to such dangerous ancestral environments was physically formidable leaders, who helped allies acquire and maintain vital resources due to their significant resource holding potential. Given an adaptive preference for physically formidable leaders, sexual dimorphism, or persistent advantages of males over females in terms of size and strength, created a non-adaptive and non-functional but lingering outcome (i.e., incidental by-product) that advantages males over females in national leadership attainment.

It is important to note that the results presented here do not fully explain or even attempt to explain “why men in all human societies have tended to wield more political leadership than women” (von Rueden et al., 2018, 403). They do, though, shed light on a high-profile and important situation in which males have had a vastly disproportionate presence: national executive leadership. Members of the polity view these national leaders as the head of the military. For instance, of the limited constitutional powers specifically given to U.S. Presidents, Article II Section 2 of the US Constitution states, “The President shall be Commander in Chief of the Army and Navy of the United States, and of the Militia of the several States” (U.S. Const. art. II, § II, 1992). When political leaders take their countries to war, the result (win, lose, or draw) affects the leader’s likelihood of remaining in office (Croco, 2011). Considerations such as culpability and vulnerability for the involvement in war affect the impact, but this does not change the fact that leaders are still held responsible for taking their country to war or coming into power during a war (Croco and Weeks, 2016). On the other hand, subnational leaders often do not have “war making” duties. As such, there may not be a link between the effects of physical formidability and gendered leadership preferences in other situations. For example, although there have been no female U.S. Presidents, there have been 44 female governors of U.S. states (Center for American Women in Politics, 2020) and in 2019 nearly 17 percent of cities with populations over 30,000 had female mayors (United States Conference of Mayors, 2020).

Methodologically, some may wonder if a demand effect is at play. That is, subjects are motivated to describe a male or physically formidable leader in the war condition because that is the most socially appropriate response irrespective of their true preferences. While this is possible in survey research, it seems unlikely here. There was little to no incentive to “respond appropriately.” Answers were not scored or tied to rewards for respondents, and the instrument was fielded online and anonymously. Further, there is little to no evidence of a demand effect for other measures. For instance, the peace and cooperation treatments did not stimulate a preference for a friendly leader relative to the war treatment (see Table 1), which some could suggest is a demand effect. Further, and more directly, in the test of the total effect, the war treatment did not stimulate a preference for male leader relative to the peace and control treatments (see Figure 2). Those effects are only detected as indirect effects (see Figure 3).

Future research needs to confirm these results through conceptual replication with different measures and varied samples, in particular samples outside Western, educated, industrialized, rich, and democratic societies, which some researchers claim are outliers on a number of characteristics and not suitable for using to generalize broadly about humans (Henrich et al., 2010). It would also be appropriate to attempt to reproduce these results using other estimation methods. For instance, Spencer et al. (2005) propose an alternative research design to assess mediated effects that they call a “measurement-of-mediation” design. This design uses a series of experiments that Spencer and colleagues suggest provides a superior approach to estimating mediation effects under certain conditions. Further, future research could advance this argument by probing the assertion that the phenomenon is an incidental by-product of the evolutionary process and not a random effect or even an adaptation. Evidence that it is a by-product or even random effect, which imply there are no implications for humans’ survival and reproduction, would suggest much different theoretical and policy considerations than evidence that it is an adaptation, which implies the implications are vital.

Despite recent strides in leadership attainment by females (Geiger and Kent, 2017), the slow progress disappoints and surprises many who recognize the leadership skills women often bring to bear on society’s pressing issues. The sluggish progress suggests that conventional explanations may be overlooking additional factors. These results along with other evidence spanning time, cultures, and species suggest these outcomes may be related to very long-term factors related to evolution that are extraordinarily difficult to overcome (Tooby and Cosmides, 1992; Li et al., 2017). If this is the case, and if some societies demand to expand the pool of leadership talent, then those societies may deem it necessary to intervene directly in democratic decision making to accelerate the expansion of their leadership pools by, for instance, implementing or increasing gender-based quotas among elected officials. Although researchers have not reached a consensus on the effects of electoral gender quotas (Dahlerup, 2012), as of 2013, 57 countries had some type of legislated gender quota for national-level legislative bodies and 37 countries had political parties with voluntary quotas (Dahlerup et al., 2013).

Regardless of what emerges on the policy agenda, this research offers a more complete explanation of the imbalance in leadership attainment between men and women. It suggests that biological factors also matter in leadership-followership behavior.

## Data Availability Statement

The raw data supporting the conclusions of this article will be made available by the authors, without undue reservation.

## Ethics Statement

The studies involving human participants were reviewed and approved by the Cooperative Congressional Election Study Human Subjects Review, Harvard University. Written informed consent for participation was not required for this study in accordance with the national legislation and the institutional requirements.

## Author Contributions

Leading explanations for advantage males have over females in national leadership attainment account for some of the variance but leave a great deal unexplained. This research evaluates the issue using the biological theory of evolution by natural selection and finds that this approach accounts for additional variance in this phenomenon. The results suggest the predominant preference for male over female leaders in some contexts may be the non-adaptive and non-functional but lingering outcome of an adaptive preference for physically formidable allies that was shaped by natural selection in ancestral environments. Both authors contributed to the article and approved the submitted version.

## Conflict of Interest

The authors declare that the research was conducted in the absence of any commercial or financial relationships that could be construed as a potential conflict of interest.

## Footnotes

1 While this article focuses on the demand for female versus male candidates, Sweet-Cushman (2016) argues evolutionary forces also affect the relative supply of female versus male candidates via differential sexual selection pressures related to the risk of running for and holding elective office. Other scholars take a broader but related approach suggesting females receive different if not smaller relative benefits from gaining status in a social hierarchy (e.g., Campbell, 1999) and likely have different measures of social status (e.g., Benenson, 1999).

## References

[B1] Aarøe L. Petersen M. B. (2013). Hunger games: fluctuations in blood glucose levels influence support for social welfare. *Psychol. Sci.* 24 2550–2556. 10.1177/0956797613495244 24171932

[B2] Adams T. G. Stewart P. A. Blanchar J. C. (2014). Disgust and the politics of sex: exposure to a disgusting odorant increases politically conservative views on sex and decreases support for gay marriage. *PLoS One* 9:e95572. 10.1371/journal.pone.0095572 24798457 PMC4010392

[B3] Alexander D. Andersen K. (1993). Gender as a factor in the attribution of leadership traits. *Polit. Res. Q.* 46 527–545. 10.2307/448946

[B4] Alford J. R. Funk C. L. Hibbing J. R. (2005). Are political orientations genetically transmitted? *Am. Polit. Sci. Rev.* 99 153–167. 10.1017/s0003055405051579

[B5] Alford J. R. Hibbing J. R. (2004). The origin of politics: an evolutionary theory of political behavior. *Perspect. Polit.* 2 707–723. 10.1017/s1537592704040460

[B6] Arceneaux K. Johnson M. Maes H. H. (2012). The genetic basis of political sophistication. *Twin Res. Hum. Genet.* 15 34–41. 10.1375/twin.15.1.34 22784451

[B7] Ashe J. Stewart K. (2012). Legislative recruitment: using diagnostic testing to explain underrepresentation. *Party Polit.* 18 687–707. 10.1177/1354068810389635

[B8] Baron R. M. Kenny D. A. (1986). The moderator–mediator variable distinction in social psychological research: conceptual, strategic, and statistical considerations. *J. Pers. Soc. Psychol.* 51 1173–1182. 10.1037/0022-3514.51.6.1173 3806354

[B9] Bauer N. M. (2015). Emotional, sensitive, and unfit for office? Gender stereotype activation and support female candidates. *Polit. Psychol.* 36 691–708. 10.1111/pops.12186

[B10] Benenson J. F. (1999). Females’ desire for status cannot be measured using male definitions. *Behav. Brain Sci.* 22 216–217. 10.1017/s0140525x99241817

[B11] Benenson J. F. (2013). The development of human female competition: allies and adversaries. *Philos. Trans. Biol. Sci.* 368:20130079. 10.1098/rstb.2013.0079 24167309 PMC3826208

[B12] Benjamin D. J. Cesarini D. van der Loos M. J. H. M. Dawes C. T. Koellinger P. D. Magnusson P. K. E. (2012). The genetic architecture of economic and political preferences. *Proc. Natl. Acad. Sci. U.S.A.* 109 8026–8031. 22566634 10.1073/pnas.1120666109 PMC3361436

[B13] Bouchard T. J. Jr. (2004). Genetic influences on human psychological traits. *Curr. Dir. Psychol. Sci.* 13 148–151.

[B14] Brown D. E. (1991). *Human Universals.* New York, NY: McGraw-Hill.

[B15] Buss D. M. Haselton M. G. Shackelford T. K. Bleske A. L. Wakefield J. C. (1998). Adaptations, exaptations, and spandrels. *Am. Psychol.* 53 533–548. 10.1037/0003-066x.53.5.533 9612136

[B16] Campbell A. (1999). Staying alive: evolution, culture, and women’s intrasexual aggression. *Behav. Brain Sci.* 22 203–214. 10.1017/s0140525x99001818 11301523

[B17] Campbell A. (2013). The evolutionary psychology of women’s aggression. *Philos. Trans. Biol. Sci.* 368 1–11. 10.1002/9781119125563.evpsych227 PMC3826207 24167308

[B18] Campbell D. E. Wolbrecht C. (2006). See jane run: women politicians as role models for adolescents. *J. Polit.* 68 233–247. 10.1111/j.1468-2508.2006.00402.x

[B19] Carey T. E. Jr. Lizotte M. K. (2019). Political experience and the intersection between race and gender. *Polit. Groups Ident.* 7 243–266. 10.1080/21565503.2017.1354036

[B20] Center for American Women in Politics (2020). *History of Women Governors.* Available online at: https://cawp.rutgers.edu/history-women-governors (accessed 09 June 2020).

[B21] Centra J. A. Gaubatz N. B. (2000). Is there gender bias in student evaluations of teaching. *J. High. Educ*. 71 17–33. 10.1080/00221546.2000.11780814

[B22] Chagnon N. A. (1997). *The Yanomamo.* London: Wadsworth.

[B23] Chagnon N. A. Irons W. (1979). *Evolutionary Biology and Human Social Behavior: An Anthropological Perspective.* North Scituate, MA: Duxbury Press.

[B24] Collier J. F. Rosaldo M. Z. (1981). “Politics and gender in simple societies,” in *Sexual Meanings: The Cultural Construction of Gender and Sexuality*, eds Sherry B. O. Whitehead H. (Cambridge: Cambridge University Press), 275–329.

[B25] Correll S. J. (2004). Constraints into preferences: gender, status, and emerging career aspirations. *Am. Sociol. Rev.* 69 93–113. 10.1177/000312240406900106

[B26] Crawford C. (2008). “Adaptations, environments, and behavior: then and now,” in *Foundations of Evolutionary Psychology*, eds Crawford C. Krebs D. (New York, NY: Lawrence Erlbaum Associates), 191–214.

[B27] Croco S. E. (2011). The decider’s dilemma: leader culpability, war outcomes, and domestic punishment. *Am. Polit. Sci. Rev.* 105 457–477. 10.1017/s0003055411000219

[B28] Croco S. E. Weeks J. (2016). War outcomes and leader tenure. *World Polit.* 68 577–607. 10.1017/s0043887116000071

[B29] Dahlerup D. (2012). *The Impact of Gender Quotas.* Oxford: Oxford University Press.

[B30] Dahlerup D. Hilal Z. Kalandadze N. Kandawasvika-Nhundu R. (2013). *Atlas of Electoral Gender Quotas.* London: IDEA.

[B31] Darwin C. (1859). *On the Origin of Species by Means of Natural Selection, or the Preservation of Favoured Races in the Struggle for Life.* London: John Murray. PMC5184128 30164232

[B32] Dawes C. Cesarini D. Fowler J. H. Johannesson M. Magnusson P. K. E. Oskarsson S. (2014). The relationship between genes, psychological traits, and political participation. *Am. J. Polit. Sci.* 58 888–903. 10.1111/ajps.12100

[B33] Dawes C. T. Settle J. E. Loewen P. J. McGue M. Iacono W. G. (2015). Genes, psychological traits and civic engagement. *Philos. Trans. R. Soc. B* 370:20150015. 10.1098/rstb.2015.0015 26503688 PMC4633851

[B34] De Neve J. E. Mikhaylov S. Dawes C. T. Christakis N. A. Fowler J. H. (2013). Born to lead? A twin design and genetic association study of leadership role occupancy. *Leadersh. Q.* 24 45–60. 10.1016/j.leaqua.2012.08.001 23459689 PMC3583370

[B35] Devitt J. (2002). Framing gender on the campaign trail: female gubernatorial candidates and the press. *J. Mass Commun. Q.* 79 445–463. 10.1177/107769900207900212

[B36] Diamond J. (1999). *Guns, Germs, and Steel: The Fates of Human Societies.* New York, NY: W. W. Norton.

[B37] Dolan K. (2010). The impact of gender stereotyped evaluations on support for women candidates. *Polit. Behav.* 32 69–88. 10.1007/s11109-009-9090-4

[B38] Dolan K. (2014). Gender stereotypes, candidate evaluations, and voting for women candidates: what really matters? *Polit. Res. Q*. 67 96–107. 10.1177/1065912913487949

[B39] Eagly A. H. Karau S. J. (2002). Role congruity theory of prejudice toward female leaders. *Psychol. Rev.* 1093 573–598. 10.1037/0033-295x.109.3.573 12088246

[B40] Eagly A. H. Nater C. Miller D. I. Kaufmann M. Sczesny S. (2019). Gender stereotypes have changed: a cross-temporal meta-analysis of US public opinion polls from 1946 to 2018. *Am. Psychol.* 75 301–315. 10.1037/amp0000494 31318237

[B41] Faulkner J. Schaller M. Park J. H. Duncan L. A. (2004). Evolved disease-avoidance mechanisms and contemporary xenophobic attitudes. *Group Processes Intergr. Relat.* 7 333–353. 10.1177/1368430204046142

[B42] Fessler D. M. T. Holbrook C. Gervais M. M. (2014). Men’s physical strength moderates conceptualizations of prospective foes in two disparate societies. *Hum. Nat.* 25 393–409. 10.1007/s12110-014-9205-4 24993128

[B43] Foley R. (1995). The adaptive legacy of human evolution: a search for the environment of evolutionary adaptedness. *Evol. Anthropol.* 4 194–203. 10.1002/evan.1360040603

[B44] Fox R. L. Lawless J. L. (2004). Entering the arena? Gender and the decision to run for office. *Am. J. Polit. Sci.* 48 264–280. 10.1111/j.0092-5853.2004.00069.x

[B45] Fox R. L. Lawless J. L. (2010). If only they’d ask: gender, recruitment, and political ambition. *J. Polit.* 72 310–326. 10.1017/s0022381609990752

[B46] French J. A. Smith K. B. Alford J. R. Guck A. Birnie A. K. Hibbing J. R. (2014). Cortisol and politics: variance in voting behavior is predicted by baseline cortisol levels. *Physiol. Behav.* 133 61–67. 10.1016/j.physbeh.2014.05.004 24835544 PMC4120245

[B47] Fulton S. A. Maestas C. D. Maisel L. S. Stone W. J. (2006). The sense of a woman: gender, ambition, and the decision to run for congress. *Polit. Res. Q.* 59 235–248. 10.1177/106591290605900206

[B48] Garfield Z. H. von Rueden C. Hagen E. H. (2019). The evolutionary anthropology of political leadership. *Leadersh. Q.* 30 59–80. 10.1016/j.leaqua.2018.09.001

[B49] Geary D. (1998). *Male, Female: The Evolution of Human Sex Differences.* Washington, DC: American Psychological Association.

[B50] Geiger A. Kent L. (2017). *Number of Women Leaders Around the World has Grown, But They’re Still a Small Group.* Washington, DC: Pew Research Center.

[B51] Giphart R. van Vugt M. (2018). *Our Stone Age Brain Deceives Us Every Day and What We Can Do about It.* London: Robinson.

[B52] Glowacki L. von Rueden C. (2015). Leadership solves collective action problems in small-scale societies. *Philos. Trans. R. Soc. B Biol. Sci.* 370:20150010. 10.1098/rstb.2015.0010 26503683 PMC4633846

[B53] Glowacki L. Wilson M. L. Wrangham R. W. (2017). The evolutionary anthropology of war. *J. Econ. Behav. Organ.* [Epub ahead of print],

[B54] Goldstein J. S. (2003). *War and Gender: How Gender Shapes the War System and Vice Versa.* Cambridge: Cambridge University Press.

[B55] Gorman E. H. (2005). Gender stereotypes, same-gender preferences, and organizational variation in the hiring of women: evidence from law firms. *Am. Sociol. Rev.* 70 702–728. 10.1177/000312240507000408

[B56] Gregor T. (1979). Short people. *Nat. Hist.* 88 14–19.

[B57] Handwerker W. P. Crosbie P. V. (1982). Sex and dominance. *Am. Anthropol.* 84 97–104.

[B58] Hatemi P. K. McDermott R. (2012). The genetics of politics: discovery, challenges, and progress. *Trends Genet.* 28 525–533. 10.1016/j.tig.2012.07.004 22951140

[B59] Hayes A. F. (2017). *Introduction to Mediation, Moderation, and Conditional Process Analysis: A Regression-Based Approach*, 2nd Edn New York, NY: Guilford Press.

[B60] Henrich J. Gil-White F. J. (2001). The evolution of prestige: freely conferred deference as a mechanism for enhancing the benefits of cultural transmission. *Evol. Hum. Behav.* 22 165–196. 10.1016/s1090-5138(00)00071-4 11384884

[B61] Henrich J. Heine S. J. Norenzayan A. (2010). The weirdest people in the world? *Behav. Brain Sci.* 33 61–83. 10.1017/s0140525x0999152x 20550733

[B62] Hensley W. E. (1993). Height as a measure of success in academe. *Psychology* 30 40–46.

[B63] Hicks R. Tingley D. (2011). Causal mediation analysis. *Stata J.* 11 605–619.

[B64] Hoehl S. Hellmer K. Johansson M. Gredebäck G. (2017). Itsy bitsy spider: infants react with increased arousal to spiders and snakes. *Front. Psychol.* 8:1710. 10.3389/fpsyg.2017.01710 29093687 PMC5651927

[B65] Huddy L. Terkildsen N. (1993). Gender stereotypes and the perception of male and female candidates. *Am. J. Polit. Sci.* 37 119–147. 10.2307/2111526

[B66] Huntingford F. A. Turner A. K. (1987). *Animal Conflict.* London: Chapman & Hall.

[B67] Iofrida C. Palumbo S. Pellegrini S. (2014). Molecular genetics and antisocial behavior: where do we stand? *Exp. Biol. Med.* 239 1514–1523. 10.1177/1535370214529508 24764243

[B68] Kahn K. F. (1992). Does being male help? An investigation of the effects of candidate gender and campaign coverage on evaluations of US Senate candidates. *J. Polit.* 54 497–517. 10.2307/2132036

[B69] Kahn K. F. (1994). Does gender make a difference? An experimental examination of sex stereotypes and press patterns in statewide campaigns. *Am. J. Polit. Sci.* 38 162–195. 10.2307/2111340

[B70] Kahn K. F. (1996). *The Political Consequences of Being a Woman: How Stereotypes Influence the Conduct of Political Campaigns.* New York, NY: Columbia University Press.

[B71] Kalin J. L. Waldron J. J. (2015). Preferences toward gender of coach and perceptions of roles of basketball coaches. *Int. J. Exer. Sci.* 8 303–317.

[B72] Kalma A. (1991). Hierarchisation and dominance assessment at first glance. *Eur. J. Soc. Psychol.* 21 165–181. 10.1002/ejsp.2420210206

[B73] Kanai R. Feilden T. Firth C. Rees G. (2011). Political orientations are correlated with brain structure in young adults. *Curr. Biol.* 21 677–680. 10.1016/j.cub.2011.03.017 21474316 PMC3092984

[B74] Kappeler P. M. Fichtel C. van Vugt M. Smith J. E. (2019). Female leadership: a transdisciplinary perspective. *Evol. Anthropol.* 28 160–163. 10.1002/evan.21783 31112358

[B75] Keegan J. (1993). *A History of Warfare.* Toronto, ON: Key Porter Books.

[B76] Keeley L. H. (1996). *War Before Civilization.* Oxford: Oxford University Press.

[B77] King D. Matland R. E. (2003). Sex and the grand old party: an experimental investigation of the effect of candidate sex on support for a republican candidate. *Am. Polit. Res.* 31 595–612. 10.1177/1532673x03255286

[B78] Klofstad C. A. (2016). Candidate voice pitch influences election outcomes. *Polit. Psychol.* 37 725–738. 10.1111/pops.12280

[B79] Koch J. (2002). Gender stereotypes and citizens’ impression of house candidates ideological orientations. *Am. J. Polit. Sci.* 46 453–462. 10.2307/3088388

[B80] Lassek W. D. Gaulin S. J. C. (2009). Costs and benefits of fat-free muscle mass in men: relationship to mating success, dietary requirements, and native immunity. *Evol. Hum. Behav.* 30 322–328. 10.1016/j.evolhumbehav.2009.04.002

[B81] Laustsen L. Petersen M. B. (2017). Perceived conflict and leader dominance: Individual and contextual factors behind preferences for dominant leaders. *Polit. Psychol.* 38 1083–1101. 10.1111/pops.12403

[B82] Lawless J. L. (2004). Women, war, and winning elections: gender stereotyping in the post-September 11th era. *Polit. Res. Q.* 57 479–490. 10.2307/3219857

[B83] Leeper M. (1991). The impact of prejudice on female candidates: an experimental look at voter inference. *Am. Polit. Q.* 19 248–261. 10.1177/1532673x9101900206

[B84] Lewis D. M. G. Al-Shawaf L. Conroy-Beam D. Asao K. Buss D. M. (2017). Evolutionary psychology: a how-to guide. *Am. Psychol.* 72 353–373. 28481582 10.1037/a0040409

[B85] Li N. P. Van Vugt M. Colarelli S. M. (2017). The evolutionary mismatch hypothesis: implications for psychological science. *Curr. Dir. Psychol. Sci.* 28:626 10.1177/0963721419885877

[B86] Little A. C. Burriss R. P. Jones B. C. Roberts S. C. (2007). Facial appearance affects voting decisions. *Evol. Hum. Behav.* 28 18–27. 10.1016/j.evolhumbehav.2006.09.002

[B87] LoBue V. DeLoache J. S. (2008). Detecting the snake in the grass: attention to fear-relevant stimuli in adults and young children. *Psychol. Sci.* 19 284–289. 10.1111/j.1467-9280.2008.02081.x 18315802

[B88] Low B. S. (1992). Sex, coalitions, and politics in preindustrial societies. *Polit. Life Sci.* 11 63–80. 10.1017/s0730938400017214

[B89] Ludwig A. M. (2002). *King of the Mountain: The Nature of Political Leadership.* Lexington, KY: University of Kentucky Press.

[B90] Lukaszewski A. W. Simmons Z. L. Anderson C. Roney J. R. (2016). The role of physical formidability in human social status allocation. *J. Pers. Soc. Psychol.* 110 385–406. 10.1037/pspi0000042 26653896

[B91] Mansbridge J. (1999). Should blacks represent blacks and women represent women? A contingent ‘Yes.’. *J. Polit.* 61 628–657. 10.2307/2647821

[B92] Manuck S. B. McCaffery J. M. (2014). Gene-environment interaction. *Annu. Rev. Psychol.* 65 41–70. 24405358 10.1146/annurev-psych-010213-115100

[B93] Matland R. E. (1994). Putting scandinavian equality to the test: an experimental evaluation of gender stereotyping of political candidates in a sample of norwegian voters. *Br. J. Polit. Sci.* 24 273–292. 10.1017/s0007123400009819

[B94] Mayr E. (2001). *What Evolution is.* New York, NY: Basic Books.

[B95] McCann S. J. H. (2001). Height, societal threat, and the victory margin in presidential elections (1824-(1992)). *Psychol. Rep.* 88 741–742. 10.2466/pr0.2001.88.3.741 11508013

[B96] McCleskey J. A. (2014). Situational, transformational, and transactional leadership and leadership development. *J. Bus. Stud. Q.* 5 117–130.

[B97] McDermott M. (1998). Race and gender cues in low-information elections. *Polit. Res. Q*. 51 895–918. 10.2307/449110

[B98] McGarty C. Yzerbyt V. Y. Spears R. (2002). “Social, cultural and cognitive factors in stereotype formation,” in *Stereotypes as Explanations: The Formation of Meaningful Beliefs about Social Groups*, eds McGarty C. Yzerbyt V. Y. Spears R. (Cambridge: Cambridge University Press), 1–15. 10.1017/cbo9780511489877.002

[B99] Merolla J. L. Burnett G. Pyle K. V. Ahmadi S. Zak P. J. (2013). Oxytocin and the biological basis for interpersonal and political trust. *Polit. Behav.* 35 753–776. 10.1007/s11109-012-9219-8

[B100] Merolla J. L. Ramos J. Zechmeister E. (2007). Crisis, charisma, and consequences: evidence from the (2004) U.S. presidential election. *J. Polit.* 69 30–42. 10.1111/j.1468-2508.2007.00492.x

[B101] Miller A. H. Wattenberg M. P. Malanchuk O. (1986). Schematic assessments of presidential candidates. *Am. Polit. Sci. Rev.* 80 521–540. 10.2307/1958272

[B102] Murray G. R. (2017). “Mass political behavior and biology,” in *Handbook of Biology and Politics*, eds Peterson S. A. Somit A. (Cheltenham: Edward Elgar Publishers), 247–261. 10.4337/9781783476275.00025

[B103] Murray G. R. (2014). Evolutionary preferences for physical formidability in leaders. *Polit. Life Sci*. 33 33–53. 10.2990/33_1_33 25514522

[B104] Murray G. R. Murray S. M. (2011). “Caveman executive leadership: evolved leadership preferences and biological sex,” in *Evolutionary Psychology in the Business Sciences*, ed. Saad G. (Cham: Springer), 135–163. 10.1007/978-3-540-92784-6_6

[B105] Nesse R. M. Williams G. C. (1994). *Why We Get Sick: The New Science of Darwinian Medicine.* New York: Vintage Books.

[B106] Niven D. (2006). Throwing your hat out of the ring: negative recruitment and the gender imbalance in state legislative candidacy. *Polit. Gender* 2 473–489.

[B107] Oskarsson S. Dawes C. T. Lindgren K. O. (2017). It runs in the family: a study of political candidacy among swedish adoptees. *Polit. Behav.* 40 883–908. 10.1007/s11109-017-9429-1 31148882 PMC6514823

[B108] Petersen M. B. Delton A. W. Robertson T. F. Tooby J. Cosmides L. (2008). “Politics of the evolved mind: political parties and coalitional reasoning,” in *Paper Presented at the 2008 Midwest Political Science Association Annual Conference*, (Chicago, IL: Midwest Political Science Association).

[B109] Petersen M. B. Laustsen L. (2020). Dominant leaders and the political psychology of followership. *Curr. Opin. Psychol.* 33 136–141. 10.1016/j.copsyc.2019.07.005 31430715

[B110] Pheasant S. T. (1983). Sex differences in strength: some observations on their variability. *Appl. Ergon.* 14 205–211. 10.1016/0003-6870(83)90083-2 15676481

[B111] Plutzer E. Zipp J. F. (1996). Identity politics, partisanship, and voting for women candidates. *Public Opin. Q.* 60 30–57. 10.1086/297738

[B112] Post C. (2015). When is female leadership an advantage? Coordination requirements, team cohesion, and team interaction norms. *J. Organ. Behav.* 36 1153–1175. 10.1002/job.2031

[B113] Preacher K. J. Hayes A. F. (2004). SPSS and SAS procedures for estimating indirect effects in simple mediation models. *Behav. Res. Methods Instr. Comput.* 36 717–731. 10.3758/bf03206553 15641418

[B114] Rosenwasser S. M. Seale J. (1988). Attitudes toward a hypothetical male or female presidential candidate – a research note. *Polit. Psychol.* 9 591–598. 10.2307/3791529

[B115] Sanbonmatsu K. (2002). Gender stereotypes and vote choice. *Am. J. Polit. Sci.* 46 20–34. 10.2307/3088412

[B116] Sanbonmatsu K. Dolan K. (2009). Do gender stereotypes transcend party? *Polit. Res. Q.* 63 485–494. 10.1177/1065912908322416

[B117] Sapiro V. (1981). If U.S. Senator baker were a woman: an experimental study of candidate images. *Polit. Psychol.* 3 61–83. 10.2307/3791285

[B118] Sapiro V. (1982). Private costs of public commitments or public costs of private commitments? Family roles versus political ambition. *Am. J. Polit. Sci.* 26 265–279. 10.2307/2111039

[B119] Sapiro V. (1983). *The Political Integration of Women: Roles, Socialization, and Politics.* Urbana: University of Illinois Press.

[B120] Scarr S. McCartney K. (1983). How people make their own environments: a theory of genotype? environment effects. *Child Dev.* 54 424–435. 10.2307/1129703 6683622

[B121] Schoenberg N. E. Ravda H. (2000). Using vignettes in awareness and attitudinal research. *Int. J. Soc. Res. Methodol.* 3 63–74. 10.1080/136455700294932

[B122] Sell A. Cosmides L. Tooby J. Sznycer D. von Rueden C. Gurven M. (2008). Human adaptations for the visual assessment of strength and fighting ability from the body and face. *Proc. R. Soc. B Biol. Sci.* 276 575–584. 10.1098/rspb.2008.1177 18945661 PMC2664345

[B123] Sell A. Hone L. S. E. Pound N. (2012). The importance of physical strength to human males. *Hum. Nat.* 23 30–44. 10.1007/s12110-012-9131-2 22477166

[B124] Shah D. V. Hanna A. Bucy E. P. Wells C. Quevedo V. (2015). The power of television images in a social media age: linking biobehavioral and computational approaches via the second screen. *Ann. Am. Acad. Polit. Soc. Sci.* 659 225–245. 10.1177/0002716215569220

[B125] Smith J. E. Ortiz C. A. Buhbe M. T. van Vugt M. (2018). Obstacles and opportunities for female leadership in mammalian societies: a comparative perspective. *Leadersh. Q.* 31:101267 10.1016/j.leaqua.2018.09.005

[B126] Smith K. Alford J. R. Hatemi P. K. Eaves L. J. Funk C. Hibbing J. R. (2012). Biology, ideology, and epistemology: how do we know political attitudes are inherited and why should we care? *Am. J. Polit. Sci.* 56 17–33. 10.1111/j.1540-5907.2011.00560.x 22400141

[B127] Spencer S. J. Zanna M. P. Fong G. T. (2005). Establishing a causal chain: why experiments are often more effective than mediational analyses in examining psychological processes. *J. Pers. Soc. Psychol.* 89 845–851. 10.1037/0022-3514.89.6.845 16393019

[B128] Spisak B. R. Dekker P. H. Krüger M. Van Vugt M. (2012). Warriors and peacekeepers: testing a biosocial implicit leadership hypothesis of intergroup relations using masculine and feminine faces. *PLoS One* 7:e30399. 10.1371/journal.pone.0030399 22276190 PMC3262824

[B129] Stadelmann D. Portman M. Eichenberger R. (2014). Politicians and preferences of the voter majority: does gender matter? *Econ. Polit.* 26 355–380. 10.1111/ecpo.12039

[B130] Stewart P. A. Bucy E. P. Mehu M. (2015). Strengthening bonds and connecting with followers: a biobehavioral inventory of political smiles. *Polit. Life Sci.* 34 73–92. 10.1017/pls.2015.5 26399947

[B131] Sweet-Cushman J. (2016). Gender, risk assessment, and political ambition. *Polit. Life Sci.* 35 1–18. 10.1017/pls.2016.13 28134044

[B132] Szamado S. (2008). “How threat displays work: species specific fighting techniques, weaponry and proximity risk.”. *Anim. Behav.* 76 1455–1463. 10.1016/j.anbehav.2008.07.010

[B133] Tooby J. Cosmides L. (1992). “Psychological foundations of culture,” in *The Adapted Mind: Evolutionary Psychology and the Generation of Culture*, eds Barlow J. Cosmides L. Tooby J. (New York: Oxford University Press), 19–136.

[B134] U.S. Const. art. II, § II (1978).

[B135] United States Conference of Mayors. (2020). *About the Women Mayors Leadership Alliance.* Available online at: https://www.usmayors.org/womens-leadership-alliance/ (accessed 09 Jun 2020)

[B136] Valentine S. (2001). Development of a brief multidimensional aversion to women who work scale. *Sex Roles* 44 773–787.

[B137] Van Vugt M. Grabo A. E. (2015). The many faces of leadership: an evolutionary-psychology approach. *Curr. Dir. Psychol. Sci.* 24 484–489. 10.1177/0963721415601971

[B138] Van Vugt M. Hogan R. Kaiser R. B. (2008a). Leadership, followership, and evolution. *Am. Psychol.* 63 182–196. 18377108 10.1037/0003-066X.63.3.182

[B139] Van Vugt M. Johnson D. D. P. Kaiser R. B. O’Gorman R. (2008b). “Evolution and the social psychology of leadership: the mismatch hypothesis,” in *Leadership at the Crossroads*, eds Forsyth D. Goethals G. R. Hoyt C. L. Genovese M. A. Han L. C. Ciulla J. B. (New York, NY: Praeger).

[B140] Van Vugt M. Spisak B. R. (2008). Sex differences in the emergence of leadership during competitions within and between groups. *Psychol. Sci*. 19 854–858. 10.1111/j.1467-9280.2008.02168.x 18947349

[B141] von Rueden C. Alami S. Kaplan H. Gurven M. (2018). Sex differences in political leadership in an egalitarian society. *Evol. Hum. Behav.* 39 402–411. 10.1016/j.evolhumbehav.2018.03.005 30319239 PMC6178229

[B142] von Rueden C. Gurven M. Kaplan H. Stieglitz J. (2014). Leadership in an egalitarian society. *Hum. Nat.* 25 538–566. 10.1007/s12110-014-9213-4 25240393 PMC4258461

[B143] Weinschenk A. C. Dawes C. T. (2017). Genes, personality traits, and the sense of civic duty. *Am. Polit. Res.* 46 47–76. 10.1177/1532673x17710760

[B144] Whyte M. K. (1978). *The Status of Women in Preindustrial Societies.* Princeton, NJ: Princeton University Press.

[B145] Zaccaro S. J. Kemp C. Bader P. (2004). “Leader traits and attributes,” in *The Nature of Leadership*, eds Antonakis J. Cianciolo A. T. Sternberg R. J. (Thousand Oaks, CA: Sage).

