# Supplementary material for: An Experimental Examination of Demand-Side Preferences for Female and Male National Leaders
Source: Front Psychol. 2020 Sep 15;11:576278. doi: 10.3389/fpsyg.2020.576278 (PMC7522344; doi:10.3389/fpsyg.2020.576278)
Supplement: Supplementary file 1 [file Data_Sheet_1.pdf]

Appendices

An Experimental Examination of Demand-Side Preferences for Female and Male National Leaders

Appendix A: Experimental Treatment Vignettes

War [CODED 1]

Imagine that your country is experiencing a time of ongoing war with neighboring countries that suggests its long-term survival is at risk. Create in your mind the national leader of your country, such as a president or prime minister, whom you would want to lead the country during a time of war. This should not be a real person but should be a fictitious person who has all the characteristics and qualities you want in the leader of your country during a time of war.

Peace [CODED 2]

Imagine that your country is experiencing a time of ongoing peace with neighboring countries that suggests its long-term survival is not at risk. Create in your mind the national leader of your country, such as a president or prime minister, whom you would want to lead the country during a time of peace. This should not be a real person but should be a fictitious person who has all the characteristics and qualities you want in the leader of your country during a time of peace.

Cooperation [CODED 3]

Imagine that your country has experienced a major natural disaster that has completely cut the supply of electricity to several million people in a number of large cities across the country. A major, nationwide cooperative effort by citizens to reduce electricity consumption is required until power can be restored to the cities. Create in your mind the national leader of your country, such as a president or prime minister, whom you would want to lead the country in this nationwide cooperative effort. This should not be a real person but should be a fictitious person who has all the characteristics and qualities you want in the leader of your country during this major, nationwide cooperative effort.

Control [CODED 4]

Create in your mind the national leader of your country, such as a president or prime minister, whom you would want to lead the country. This should not be a real person but should be a fictitious person who has all the characteristics and qualities you want in the leader of your country.

Appendix B: Model for Randomization Check Results

This multinomial probit test of random assignment to the experimental groups indicates the randomization process generated statistically equivalent experimental groups ($X^2[69] = 47.38$, $p = 0.98$). In this test, group assignment was regressed on subject gender stereotyping, political ideology, income, education, race, gender, age, religiosity, and political interest.

Variables by Treatment Group	Coef	Robust SE	P>|z|	95% CI	
1_War					
Stereotyping	-0.030	0.362	0.934	-0.738	0.679
Female	-0.056	0.143	0.693	-0.336	0.223
Age	-0.004	0.005	0.406	-0.014	0.005
Ideology					
1/mod	0.005	0.188	0.978	-0.363	0.373
2/con	0.077	0.181	0.673	-0.279	0.432
99/NotSure	-0.172	0.382	0.653	-0.922	0.577
Income					
2/Qrtl_2	0.291	0.210	0.166	-0.120	0.702
3/Qrtl_3	0.159	0.204	0.435	-0.240	0.559
4/Qrtl_4	0.362	0.210	0.085	-0.050	0.773
99/miss	0.126	0.245	0.608	-0.354	0.605
Race/Ethnicity					
2/Black	0.318	0.217	0.144	-0.108	0.744
3/Hispanic	-0.040	0.263	0.880	-0.556	0.476
6/Mix	-0.161	0.424	0.704	-0.992	0.670
99/Other	-0.667	0.355	0.061	-1.363	0.030
Education					
2/Some Coll	0.265	0.173	0.125	-0.073	0.603
3/Coll Degree	0.347	0.186	0.062	-0.017	0.711
Political Interest					
2/OnlyNowThen	0.046	0.341	0.892	-0.622	0.715
3/Sometimes	-0.254	0.313	0.418	-0.868	0.360
4/Most Times	-0.156	0.320	0.625	-0.783	0.471
99/miss	0.417	0.611	0.495	-0.781	1.615
Religion					
Somewhat	0.106	0.204	0.603	-0.294	0.506
Not much	0.208	0.220	0.344	-0.223	0.639
Not at all	0.078	0.226	0.732	-0.366	0.521
Constant	-0.229	0.494	0.643	-1.197	0.738

2_Peace					
Stereotyping	-0.030	0.354	0.933	-0.723	0.663
Female	0.066	0.141	0.639	-0.211	0.343
Age	-0.001	0.005	0.821	-0.010	0.008
Ideology					
1/mod	-0.099	0.187	0.597	-0.464	0.267
2/con	-0.122	0.181	0.503	-0.477	0.234
99/NotSure	0.050	0.347	0.885	-0.631	0.731
Income					
2/Qrtl_2	0.198	0.206	0.338	-0.207	0.602
3/Qrtl_3	0.179	0.197	0.366	-0.208	0.565
4/Qrtl_4	0.215	0.209	0.305	-0.196	0.625
99/miss	0.075	0.236	0.750	-0.387	0.537
Race/Ethnicity					
2/Black	0.227	0.213	0.286	-0.190	0.644
3/Hispanic	-0.052	0.260	0.842	-0.561	0.457
6/Mix	-0.795	0.468	0.090	-1.713	0.123
99/Other	-0.518	0.346	0.134	-1.195	0.159
Education					
2/Some Coll	0.203	0.170	0.232	-0.130	0.536
3/Coll Degree	0.311	0.184	0.091	-0.050	0.671
Political Interest					
2/OnlyNowThen	0.302	0.339	0.372	-0.362	0.966
3/Sometimes	-0.060	0.310	0.846	-0.667	0.547
4/Most Times	0.035	0.314	0.910	-0.580	0.651
99/miss	-0.177	0.666	0.791	-1.482	1.129
Religion					
Somewhat	-0.008	0.196	0.969	-0.392	0.376
Not much	-0.045	0.211	0.830	-0.458	0.368
Not at all	-0.260	0.220	0.237	-0.690	0.171
Constant	-0.148	0.494	0.765	-1.117	0.821
3_Cooperation	(base)				
4_Control					
Stereotyping	-0.020	0.362	0.956	-0.729	0.689
Female	0.107	0.142	0.453	-0.172	0.385
Age	-0.002	0.005	0.689	-0.011	0.008
Ideology					
1/mod	-0.074	0.187	0.690	-0.440	0.291
2/con	0.029	0.179	0.871	-0.321	0.379
99/NotSure	0.116	0.353	0.742	-0.575	0.807
Income					

2/Qrtl_2	0.384	0.206	0.062	-0.019	0.787
3/Qrtl_3	0.251	0.198	0.205	-0.137	0.639
4/Qrtl_4	0.395	0.208	0.057	-0.012	0.802
99/miss	0.080	0.242	0.741	-0.395	0.555
Race/Ethnicity					
2/Black	-0.022	0.222	0.919	-0.457	0.412
3/Hispanic	0.252	0.250	0.314	-0.238	0.743
6/Mix	-0.285	0.414	0.490	-1.096	0.526
99/Other	-0.317	0.331	0.339	-0.966	0.332
Education					
2/Some Coll	0.201	0.171	0.241	-0.134	0.535
3/Coll Degree	0.277	0.185	0.134	-0.085	0.639
Political Interest					
2/OnlyNowThen	0.600	0.359	0.094	-0.103	1.304
3/Sometimes	0.236	0.332	0.478	-0.416	0.887
4/Most Times	0.346	0.339	0.307	-0.318	1.010
99/miss	0.773	0.609	0.204	-0.421	1.966
Religion					
Somewhat	0.121	0.201	0.547	-0.272	0.514
Not much	0.081	0.216	0.708	-0.343	0.505
Not at all	0.138	0.219	0.528	-0.291	0.567
Constant	-0.777	0.519	0.134	-1.793	0.239
N	977				
Wald χ^2 (69)	47.38		0.978		
Log Pseudo Like	-1330.44				

Appendix C: Models for Figure 2, Results from the Underlying Experiment and Total Effects

						Model		
	Coef	SE	P>|z|	95% CI		N	P χ^2	Pseudo R^2
War v. Cntl	0.150	0.144	0.296	-0.132	0.432	433	0.296	0.003
Constant	0.890	0.097	0.000	0.700	1.080			
dy/dx	*0.038*	*0.036*	*0.296*	*-0.033*	*0.108*			
War v. Peace	0.146	0.145	0.313	-0.137	0.430	429	0.313	0.003
Constant	0.895	0.098	0.000	0.703	1.086			
dy/dx	*0.037*	*0.036*	*0.312*	*-0.034*	*0.107*			
War v. Coop	0.294	0.141	0.037	0.017	0.571	432	0.038	0.010
Constant	0.747	0.093	0.000	0.564	0.929			
dy/dx	*0.079*	*0.038*	*0.036*	*0.005*	*0.153*			

Dependent Variable = Male Leader (1), Female Leader (0); probit regression.

Appendix D: Models for Table 1 (Treatments Stimulate Preferences for Leader Characteristics)

War v. Control

							Model		
DV	IV	Coef	SE	P>|z|	95% CI		N	P χ^2	Pseudo R^2
Athletic	War v Cntl	0.015	0.139	0.913	-0.259	0.289	451	0.913	0.000
	Constant	4.793	0.094	0.000	4.609	4.978			
Attractive	War v Cntl	-0.012	0.142	0.933	-0.291	0.267	443	0.933	0.000
	Constant	4.674	0.093	0.000	4.492	4.856			
Competent	War v Cntl	0.092	0.106	0.384	-0.116	0.299	448	0.385	0.002
	Constant	6.403	0.075	0.000	6.255	6.551			
Dependable	War v Cntl	0.085	0.094	0.369	-0.100	0.269	453	0.369	0.002
	Constant	6.444	0.069	0.000	6.308	6.579			
Dominant	War v Cntl	0.244	0.141	0.085	-0.034	0.522	449	0.085	0.007
	Constant	4.958	0.100	0.000	4.761	5.154			
Friendly	War v Cntl	-0.074	0.117	0.528	-0.303	0.156	455	0.528	0.001
	Constant	5.971	0.078	0.000	5.818	6.124			
Intelligent	War v Cntl	0.158	0.096	0.100	-0.030	0.347	451	0.100	0.006
	Constant	6.428	0.073	0.000	6.284	6.572			
Phys Fit	War v Cntl	0.162	0.136	0.233	-0.105	0.430	448	0.233	0.003
	Constant	5.433	0.095	0.000	5.247	5.619			
Phys Impose	War v Cntl	0.436	0.167	0.009	0.108	0.765	448	0.009	0.015
	Constant	3.729	0.109	0.000	3.515	3.943			
Phys Strong	War v Cntl	0.198	0.141	0.160	-0.078	0.475	447	0.160	0.005
	Constant	4.915	0.091	0.000	4.737	5.094			
Scales									
Phys Form	War v Cntl	0.328	0.132	0.014	0.067	0.588	440	0.014	0.014
	Constant	4.319	0.082	0.000	4.157	4.481			
Classic	War v Cntl	0.117	0.089	0.192	-0.059	0.292	458	0.192	0.004
	Constant	6.424	0.067	0.000	6.294	6.555			

(Appendix D, cont)

War v. Peace

DV	IV	Coef	SE	P>|z|	95% CI		Model N	Model P χ^2	Model Pseudo R^2
Athletic	War v Pce	0.016	0.142	0.908	-0.262	0.294	440	0.908	0.000
	Constant	4.792	0.097	0.000	4.601	4.983			
Attractive	War v Pce	-0.077	0.144	0.592	-0.359	0.205	436	0.592	0.001
	Constant	4.739	0.095	0.000	4.552	4.925			
Competent	War v Pce	0.032	0.104	0.756	-0.172	0.237	439	0.756	0.000
	Constant	6.463	0.073	0.000	6.319	6.607			
Dependable	War v Pce	0.052	0.095	0.582	-0.134	0.238	443	0.583	0.001
	Constant	6.476	0.070	0.000	6.339	6.613			
Dominant	War v Pce	0.400	0.142	0.005	0.121	0.679	440	0.005	0.018
	Constant	4.802	0.101	0.000	4.604	5.000			
Friendly	War v Pce	-0.168	0.115	0.147	-0.395	0.059	445	0.147	0.005
	Constant	6.065	0.075	0.000	5.917	6.213			
Intelligent	War v Pce	0.039	0.089	0.666	-0.137	0.214	447	0.666	0.000
	Constant	6.547	0.065	0.000	6.420	6.674			
Phys Fit	War v Pce	-0.073	0.128	0.570	-0.325	0.179	439	0.570	0.001
	Constant	5.668	0.083	0.000	5.506	5.831			
Phys Impose	War v Pce	0.362	0.171	0.035	0.025	0.698	441	0.036	0.010
	Constant	3.803	0.115	0.000	3.577	4.030			
Phys Strong	War v Pce	0.031	0.143	0.826	-0.250	0.313	442	0.826	0.000
	Constant	5.082	0.094	0.000	4.897	5.267			
Scales									
Phys Form	War v Pce	0.215	0.136	0.114	-0.052	0.481	436	0.114	0.006
	Constant	4.432	0.087	0.000	4.261	4.603			
Classic	War v Pce	0.055	0.088	0.532	-0.118	0.228	452	0.532	0.001
	Constant	6.486	0.065	0.000	6.359	6.613			

(Appendix D, cont)

War v. Cooperation

DV	IV	Coef	SE	P>|z|	95% CI		Model N	Model P χ²	Model Pseudo R^2
Athletic	War v Coop	0.161	0.147	0.273	-0.127	0.449	441	0.273	0.003
	Constant	4.648	0.104	0.000	4.443	4.853			
Attractive	War v Coop	0.164	0.150	0.276	-0.131	0.460	437	0.276	0.003
	Constant	4.498	0.105	0.000	4.292	4.704			
Competent	War v Coop	0.121	0.105	0.250	-0.086	0.328	440	0.250	0.003
	Constant	6.374	0.075	0.000	6.227	6.521			
Dependable	War v Coop	0.085	0.096	0.377	-0.104	0.274	442	0.377	0.002
	Constant	6.443	0.072	0.000	6.301	6.584			
Dominant	War v Coop	0.424	0.143	0.003	0.142	0.705	443	0.003	0.019
	Constant	4.778	0.103	0.000	4.576	4.981			
Friendly	War v Coop	-0.063	0.122	0.606	-0.303	0.177	441	0.606	0.001
	Constant	5.960	0.085	0.000	5.792	6.128			
Intelligent	War v Coop	0.140	0.089	0.118	-0.036	0.316	446	0.118	0.006
	Constant	6.446	0.065	0.000	6.319	6.573			
Phys Fit	War v Coop	0.141	0.136	0.300	-0.127	0.409	437	0.300	0.003
	Constant	5.454	0.095	0.000	5.267	5.640			
Phys Impose	War v Coop	0.469	0.171	0.006	0.133	0.805	439	0.006	0.017
	Constant	3.696	0.114	0.000	3.471	3.921			
Phys Strong	War v Coop	0.255	0.149	0.089	-0.039	0.548	438	0.089	0.007
	Constant	4.859	0.103	0.000	4.656	5.062			
Scales									
Phys Form	War v Coop	0.367	0.139	0.009	0.093	0.641	430	0.009	0.016
	Constant	4.279	0.093	0.000	4.096	4.462			
Classic	War v Coop	0.116	0.087	0.182	-0.055	0.288	450	0.182	0.004
	Constant	6.425	0.064	0.000	6.299	6.550			

Appendix E: Descriptions of Imagined Leader and Other Selected Measures

In the box below, describe your national leader by entering as many details as possible about this person in response to the following questions.

A. What are the leader's _political_ characteristics and qualities?

b. What are the leader's _personal_ characteristics and qualities?

c. What are the leader's _physical_ characteristics?

So we can get as accurate a description as possible of your preferred leader, please answer the following questions.

What is the gender of the leader you described?

Note that the instrument also asked about the leader’s other demographics such as age, education, income, and marital status.

STEREOTYPING SCALE

“Aversion to Women Who Work Scale” or work equality scale (Valentine 2001): A 10-item measure of aversion to women who work or, in particular, employment-related gender attitudes. Scaled 1-7 where 1=agree strongly. The instructions read:

> In talking to people about the role of women in society, we often find there are many people with more traditional views and there are many people with less traditional views. Here are a number of statements about women in society. Please indicate the extent to which you agree or disagree with each statement.

Items:

1. Women lack the skills and abilities needed at work.
2. Women are not suited for work outside of the home.
3. I am skeptical about women’s effectiveness in the workplace.
4. Women’s personal characteristics make life at work difficult.
5. Women frequently find the demands of work difficult.
6. Traditional husband/wife roles are the best.
7. Women are happier in traditional roles.
8. A woman’s place is in the home.
9. An employed wife leads to juvenile delinquency.
10. Women with families do not have time for other employment.

Appendix F: Models for Table 2 (Preferences for Male Leader, by Physically Formidable Leader, Stereotyping, and Covariates)

						Model		
Model/Column 1	Coef	SE	P>|z|	95% CI		N	P χ^2	Pseudo R^2
Phys Form	1.253	0.242	0.000	0.779	1.727	826	0.000	0.038
Constant	0.196	0.139	0.158	-0.076	0.468			
dy/dx								
Phys Form	*0.325*	*0.060*	*0.000*	*0.207*	*0.443*			
Model/Column 2								
Phys Form	1.265	0.252	0.000	0.771	1.758	826	0.000	0.065
Stereotyping	1.225	0.327	0.000	0.584	1.866			
Constant	-0.054	0.161	0.738	-0.369	0.261			
dy/dx								
Phys Form	*0.320*	*0.060*	*0.000*	*0.201*	*0.438*			
Stereotyping	*0.310*	*0.080*	*0.000*	*0.153*	*0.466*			
Model/Column 3								
Phys Form	1.281	0.252	0.000	0.787	1.774	826	0.000	0.110
Stereotyping	0.906	0.328	0.006	0.264	1.548			
Female	-0.376	0.113	0.001	-0.598	-0.154			
Age	0.007	0.003	0.039	0.000	0.013			
Education								
2/SomeColl	-0.188	0.139	0.176	-0.460	0.084			
3/CollDeg	0.000	0.144	0.999	-0.281	0.282			
Religion								
Somewhat	0.217	0.168	0.197	-0.113	0.547			
Not Much	-0.115	0.169	0.496	-0.447	0.217			
Not at All	-0.359	0.171	0.036	-0.693	-0.024			
Constant	0.010	0.312	0.974	-0.601	0.621			
dy/dx								
Phys Form	*0.307*	*0.058*	*0.000*	*0.193*	*0.420*			
Stereotyping	*0.217*	*0.077*	*0.005*	*0.066*	*0.369*			
Female	*-0.090*	*0.027*	*0.001*	*-0.143*	*-0.037*			
Age	*0.002*	*0.001*	*0.038*	*0.000*	*0.003*			
Education								
2/SomeColl	*-0.046*	*0.033*	*0.168*	*-0.111*	*0.019*			
3/CollDeg	*0.000*	*0.032*	*0.999*	*-0.063*	*0.064*			
Religion								
Somewhat	*0.046*	*0.037*	*0.213*	*-0.026*	*0.119*			
Not Much	*-0.028*	*0.041*	*0.491*	*-0.109*	*0.052*			
Not at All	*-0.096*	*0.045*	*0.030*	*-0.184*	*-0.009*			

Model/Column 4								
Phys Form	1.301	0.256	0.000	0.799	1.802	826	0.000	0.141
Stereotyping	0.524	0.333	0.116	-0.130	1.177			
Female	-0.365	0.117	0.002	-0.594	-0.135			
Age	0.003	0.004	0.396	-0.004	0.010			
Education								
2/SomeColl	-0.187	0.141	0.185	-0.462	0.089			
3/CollDeg	0.024	0.149	0.870	-0.268	0.317			
Religion								
Somewhat	0.307	0.176	0.081	-0.037	0.651			
Not Much	0.009	0.177	0.961	-0.338	0.356			
Not at All	-0.184	0.184	0.316	-0.545	0.176			
Ideology								
1/mod	0.196	0.143	0.170	-0.084	0.476			
2/con	0.667	0.155	0.000	0.362	0.971			
99/not sure	-0.146	0.245	0.553	-0.626	0.335			
Constant	-0.116	0.325	0.720	-0.752	0.520			
dy/dx								
Phys Form	0.300	0.056	0.000	0.190	0.411			
Stereotyping	0.121	0.077	0.115	-0.029	0.271			
Female	-0.084	0.027	0.002	-0.137	-0.031			
Age	0.001	0.001	0.395	-0.001	0.002			
Education								
2/SomeColl	-0.044	0.033	0.177	-0.108	0.020			
3/CollDeg	0.005	0.032	0.870	-0.058	0.069			
Religion								
Somewhat	0.066	0.040	0.094	-0.011	0.144			
Not Much	0.002	0.043	0.961	-0.082	0.086			
Not at All	-0.048	0.047	0.311	-0.141	0.045			
Ideology								
1/mod	0.054	0.039	0.168	-0.023	0.131			
2/con	0.154	0.036	0.000	0.083	0.225			
99/not sure	-0.045	0.077	0.563	-0.196	0.107			

Dependent Variable = Male Leader (1), Female Leader (0); probit regression.

Appendix G: Models for Figure 3, Mediation Analyses Results

War v. Control

	Coef	SE	P>|z|	95% CI		Model N	Model P	Model R^2
War v. Control	0.060	0.022	0.008	0.016	0.103	411	0.008	0.015
Constant	0.559	0.015	0.000	0.528	0.589			
War v. Control	0.053	0.163	0.744	-0.267	0.374	411	0.000	0.171
Phys Form	1.552	0.357	0.000	0.852	2.252			
Stereotyping	0.371	0.459	0.420	-0.530	1.271			
Female	-0.219	0.171	0.201	-0.555	0.116			
Age	0.006	0.005	0.245	-0.004	0.017			
Education								
2/SomeColl	0.008	0.218	0.972	-0.419	0.434			
3/CollDeg	0.056	0.219	0.799	-0.374	0.485			
Religion								
Somewhat	0.159	0.273	0.560	-0.376	0.694			
Not Much	-0.061	0.276	0.826	-0.603	0.481			
Not at All	-0.039	0.282	0.889	-0.592	0.513			
Ideology								
1/mod	0.365	0.215	0.090	-0.057	0.786			
2/con	0.795	0.227	0.000	0.350	1.239			
99/not sure	-0.645	0.365	0.078	-1.360	0.071			
Constant	-0.517	0.499	0.301	-1.495	0.462			

Effect	Mean	P>|z|	95% CI	
Average Mediation	0.019	0.022	0.004	0.037
Average Direct Effect	0.011		-0.059	0.082
% of Tot Eff Mediated	0.405		-5.947	9.629

(Appendix G, cont)

War v. Peace

	Coef	SE	P>|z|	95% CI		Model N	Model P	Model R^2
War v. Peace	0.041	0.023	0.076	-0.004	0.085	407	0.076	0.005
Constant	0.578	0.016	0.000	0.546	0.609			
War v. Peace	0.056	0.166	0.737	-0.270	0.381	407	0.000	0.184
Phys Form	1.832	0.368	0.000	1.110	2.553			
Stereotyping	0.395	0.487	0.418	-0.560	1.350			
Female	-0.420	0.180	0.020	-0.772	-0.068			
Age	0.002	0.006	0.774	-0.009	0.012			
Education								
2/SomeColl	-0.386	0.180	0.096	-0.841	0.069			
3/CollDeg	-0.089	0.232	0.715	-0.569	0.390			
Religion								
Somewhat	0.259	0.245	0.332	-0.265	0.782			
Not Much	-0.119	0.267	0.661	-0.649	0.411			
Not at All	-0.196	0.270	0.476	-0.735	0.343			
Ideology								
1/mod	0.136	0.275	0.521	-0.280	0.553			
2/con	0.675	0.212	0.003	0.231	1.119			
99/not sure	-0.409	0.226	0.274	-1.143	0.324			
Constant	-0.018	0.550	0.974	-1.096	1.060			

Effect	Mean		P>|z|	95% CI	
Average Mediation	0.015		0.104	-0.002	0.035
Average Direct Effect	0.011			-0.058	0.081
% of Tot Eff Mediated	0.317			-4.076	4.842

(Appendix G, cont)

War v. Cooperation

	Coef	SE	P>|z|	95% CI		Model N	Model P	Model R^2
War v. Cooperation	0.060	0.023	0.010	0.014	0.106	404	0.010	0.014
Constant	0.558	0.016	0.000	0.526	0.590			
War v. Cooperation	0.312	0.161	0.053	-0.004	0.628	404	0.000	0.170
Phys Form	1.613	0.344	0.000	0.939	2.287			
Stereotyping	1.327	0.491	0.007	0.365	2.289			
Female	-0.211	0.165	0.203	-0.535	0.113			
Age	0.005	0.005	0.362	-0.005	0.015			
Education								
2/SomeColl	-0.157	0.203	0.438	-0.554	0.240			
3/CollDeg	0.171	0.222	0.441	-0.265	0.608			
Religion								
Somewhat	0.407	0.251	0.104	-0.084	0.898			
Not Much	0.268	0.254	0.293	-0.231	0.766			
Not at All	0.003	0.258	0.990	-0.503	0.510			
Ideology								
1/mod	0.463	0.212	0.029	0.048	0.877			
2/con	0.665	0.206	0.001	0.261	1.070			
99/not sure	0.061	0.363	0.866	-0.650	0.772			
Constant	-1.090	0.504	0.030	-2.078	-0.103			
Effect	Mean		P>|z|	95% CI				
Average Mediation	0.022		0.025	0.004	0.042			
Average Direct Effect	0.070			-0.006	0.143			
% of Tot Eff Mediated	0.236			0.127	1.165			
